# The Complete *Campylobacter jejuni* Transcriptome during Colonization of a Natural Host Determined by RNAseq

**DOI:** 10.1371/journal.pone.0073586

**Published:** 2013-08-21

**Authors:** Michael E. Taveirne, Casey M. Theriot, Jonathan Livny, Victor J. DiRita

**Affiliations:** 1 Department of Microbiology and Immunology, University of Michigan Medical School, Ann Arbor, Michigan, United States of America; 2 Department of Internal Medicine, Division of Infectious Diseases, University of Michigan, Ann Arbor, Michigan, United States of America; 3 Department of Internal Medicine, Division of Pulmonary and Critical Care, University of Michigan, Ann Arbor, Michigan, United States of America; 4 Genome Sequencing and Analysis Program, Broad Institute, Cambridge, Massachusetts, United States of America; University of Maryland School of Medicine, United States of America

## Abstract

*Campylobacter jejuni* is a major human pathogen and a leading cause of bacterial derived gastroenteritis worldwide. *C. jejuni* regulates gene expression under various environmental conditions and stresses, indicative of its ability to survive in diverse niches. Despite this ability to highly regulate gene transcription, *C. jejuni* encodes few transcription factors and its genome lacks many canonical transcriptional regulators. High throughput deep sequencing of mRNA transcripts (termed RNAseq) has been used to study the transcriptome of many different organisms, including *C. jejuni*; however, this technology has yet to be applied to defining the transcriptome of *C. jejuni* during *in vivo* colonization of its natural host, the chicken. In addition to its use in profiling the abundance of annotated genes, RNAseq is a powerful tool for identifying and quantifying, as-of-yet, unknown transcripts including non-coding regulatory RNAs, 5’ untranslated regulatory elements, and anti-sense transcripts. Here we report the complete transcriptome of *C. jejuni* during colonization of the chicken cecum and in two different *in vitro* growth phases using strand-specific RNAseq. Through this study, we identified over 250 genes differentially expressed *in vivo* in addition to numerous putative regulatory RNAs, including trans-acting non-coding RNAs and anti-sense transcripts. These latter potential regulatory elements were not identified in two prior studies using ORF-based microarrays, highlighting the power and value of the RNAseq approach. Our results provide new insights into how *C. jejuni* responds and adapts to the cecal environment and reveals new functions involved in colonization of its natural host.

## Introduction

*Campylobacter jejuni* is an important human pathogen and a leading cause of bacterial derived gastroenteritis worldwide [1,2]. Human *Campylobacter* infections are sporadic foodborne diseases commonly associated with foods of animal origin, with *C. jejuni* being the predominant species associated with human illness [3]. Preventative measures to limit human exposure have focused on identifying genes and loci required for colonization [4–8] as these represent targets for anti- *Campylobacter* strategies. Although much research has been carried out characterizing regulatory mechanisms in this human pathogen, most of this work has focused on *in vitro* studies with little knowledge generated towards understanding the mechanisms of global gene expression during colonization.

The five *C. jejuni* strains sequenced to date contain approximately 1,650 to 1,800 protein-encoding genes [9–11]. *C. jejuni* highly regulates gene expression under various stresses and environmental conditions [4,12–18]. Despite its ability to rapidly alter gene expression, *C. jejuni* encodes only three sigma factors and a total of 34 other identified transcriptional regulators [9,10]. Furthermore, *C. jejuni* lacks homologues of many canonical transcriptional regulators such as FNR, CRP, OxyR, RpoS, SoxRS and ArcA [9,10]. Although there appears to be a paucity of encoded transcription factors, other mechanisms of gene regulation have been identified in *C. jejuni*. Slip-strand mispairing, leading to phase variation gene expression, has been demonstrated to be a regulatory mechanism of flagella biosynthesis [19,20], lipooligosaccharide glycosylation [21], and capsule production [22]. Furthermore, genome rearrangements [23], interspecies genetic exchange [24] and modification of RNA polymerase [25,26] are alternative mechanisms of genetic regulation employed by *C. jejuni*.

Regulatory networks in *C. jejuni* have been characterized principally through *in vitro* methods including quantitative reverse transcriptase PCR (qRT-PCR), transcriptional reporter strains and microarray studies. These have yielded tremendous amounts of information as to how this organism regulates gene expression; however, many of these studies focused on regulatory mechanisms of specific genes and loci in response to specific substrates and culture conditions *in vitro*. Only two studies have evaluated the transcriptome of *C. jejuni* during *in vivo* colonization of an animal model, including the day-of-hatch chicken model [18] and the rabbit ileal loop model [27]. These studies, utilizing microarray approaches, determined that there are marked differences in gene expression profiles between *in vivo* and *in vitro* samples, illustrating the importance for continued research into how *C. jejuni* regulates gene expression during colonization.

High throughput sequencing of cDNA libraries (RNAseq) has emerged as a powerful approach for mapping transcriptomes and profiling gene expression in diverse bacteria [28–32]. RNAseq has several key advantages over microarray analysis, including 1) the ability to detect and quantify transcripts derived from all regions of the genome, 2) a large dynamic range that affords high sensitivity for low-abundance transcripts, and 3) single nucleotide resolution [33].

Regulatory mechanisms involving small, non-coding RNAs (ncRNAs), or riboswitches have not been well-characterized in *C. jejuni*. Recently, two *in vitro* RNAseq studies mapped the transcriptome of *C. jejuni* and other *Campylobacter* species [29,30]. One study characterized the regulon of the sigma factor RpoN and correlated transcriptome expression data with protein expression profiles [29]. In the other study, RNAseq was used to map and compare transcriptional start sites, characterize promoter structures and analyze CRISPR RNAs in multiple *Campylobacter* strains and species [30]. Moreover, both studies identified a wide repertoire of potential non-coding RNA species. These *in vitro* RNAseq studies have yielded much information into regulatory mechanisms encoded by *Campylobacter* species; however, this powerful tool has yet to be applied to characterizing the transcriptome of *C. jejuni* during colonization of its natural host. Although, *C. jejuni* encodes non-coding RNA species, it does not encode the conserved RNA binding protein Hfq [9,10], a key component of sRNA regulatory mechanisms in other organisms [34–37]. *C. jejuni* does, however, encode another conserved RNA binding protein CsrA [9,10] which, in other organisms, plays a role in regulating many processes including motility [38], virulence [39], biofilm formation and central carbon metabolism [40]. The *C. jejuni* CsrA homologue is involved in the oxidative stress response, biofilm formation, and host cell invasion [41]. Unlike other microbes that use CsrA, *C. jejuni* does not encode any obvious homologue of the CsrA-antagonizing small RNA *csrB*; thus the mechanism of CsrA regulation in this pathogen is unknown. Although the regulatory mechanisms of non-coding RNAs have yet to be characterized in *C. jejuni*, the genome of the closely related organism *Helicobacter pylori* (which like *Campylobacter* encodes CsrA but not Hfq), encodes non-coding RNAs as well as riboswitches and antisense transcripts involved in regulatory processes [32,42], suggesting *C. jejuni* may also regulate gene expression with non-coding RNAs.

Here we report the first complete transcriptome of *C. jejuni* during *in vivo* colonization of the chicken cecum using RNAseq. We used a strand-specific, library construction protocol to identify both sense and antisense transcripts. Through this study we identified 272 genes that are differentially expressed *in vivo* compared to *in vitro* mid-log and stationary phase grown cultures and identified 51 potential RNA regulators including small, non-coding RNAs (ncRNAs), anti-sense transcripts and probable riboswitches. We also identified the structure of the chick cecal microbiome and identified differences in microbiome structure in *C. jejuni* colonized chicks compared to mock infected controls. Overall, we identified *in vivo* expression patterns and possible uncharacterized regulatory mechanisms that provide insight into how *C. jejuni* is able to adapt to diverse environments.

## Results and Discussion

### Mapping the in vivo and in vitro transcriptome of C. jejuni using Illumina-based RNAseq

**Figure 1:**
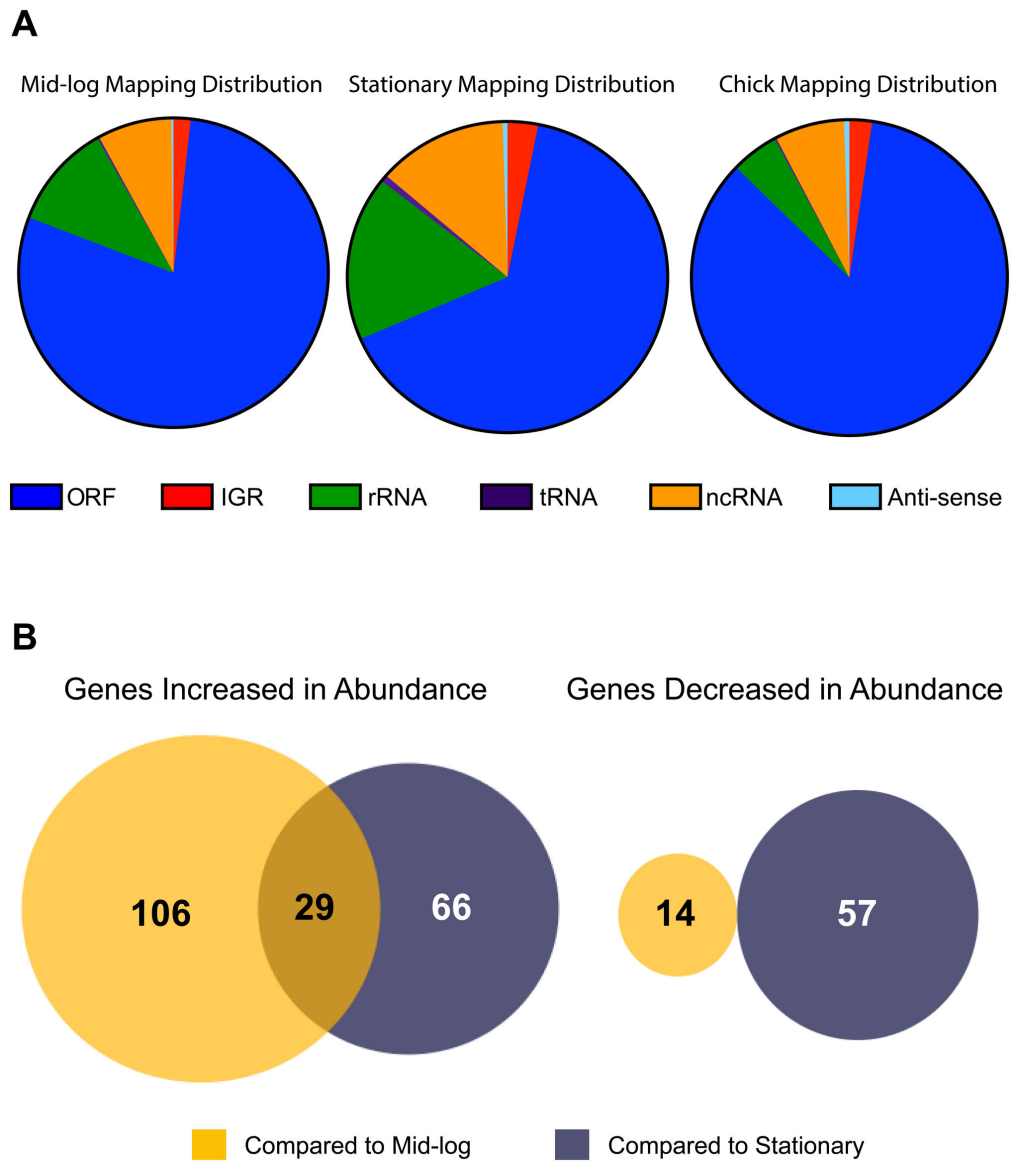
Mapping and expression profiles of *in vitro* and *in vivo* sequence reads to the *C. jejuni* 81-176 genome. A. Distribution of reads (as percent of total reads) that mapped back to specific locations of the *C. jejuni* 81-176 genome. B. Venn diagram of genes with increased and decreased abundance *in vivo* compared to *in vitro* samples. ORF: open reading frame, IGR: intergenic region, rRNA: ribosomal RNA, tRNA: transfer RNA, ncRNA: non-coding RNA, Anti-sense: anti-sense RNA.

*C. jejuni* gene expression is highly regulated; however, the mechanisms of this regulation are still largely uncharacterized. To profile the *C. jejuni* transcriptome *in vivo*, RNA was isolated from the cecal contents of chicks seven days post colonization with an average of ~7.5 x 10^8^ *C. jejuni* CFU/g cecal contents. To generate enough starting RNA for library construction (5 µg of DNA-free RNA), RNA isolated from five separate chicks housed in the same brooder, was pooled together. In total, three pools of RNA were isolated from three separate sets of chicks. *Campylobacter* strand specific, bar-coded cDNA libraries were generated using methods described by Mandlik et al. [31]. Bar-coded cDNA libraries were also generated from three independent cultures of *C. jejuni* grown in Mueller Hinton Broth (MHB) to mid-log and stationary phase, respectively. The six *in vitro* libraries were sequenced on a total of two lanes of the Illumina HiSeq2000 platform; each of the three *in vivo* samples was sequenced in its own lane. The average number of reads obtained for *in vitro* samples was ~125.4 million reads per sample, with ~87.2% of reads mapping to the *C. jejuni* 81-176 chromosome and two plasmids, pVir and pTet (Table 1). Sequencing of the *in vivo* samples resulted in ~378.8 million reads per sample, in which ~14.6% of reads mapped to the *C. jejuni* chromosome and plasmids (Table 1). Most reads mapped to open reading frames; however, many reads mapped to intergenic non-coding regions on the chromosome as well as locations anti-sense to open reading frames (ORFs), suggesting that *C. jejuni* encodes ncRNAs that could be involved in uncharacterized regulatory mechanisms (Figure 1A).

**Table 1:** Summary of sequencing results of *in vitro* and *in vivo* Illumina based cDNA libraries.

Growth Condition	Sample #	Number of Reads (in millions)	% Reads Aligned to Genome	% Reads Aligned in Pairs
	1	111.72	90.21	80.24
**Mid-Log**	2	149.09	90.87	82.90
	3	106.84	89.61	80.52
	1	114.91	84.14	76.22
**Stationary**	2	112.21	83.53	74.01
	3	157.25	84.33	75.51
	1	389.47	15.34	89.58
**Chick**	2	379.03	11.17	87.63
	3	367.79	17.29	85.56

### Structural differences in the chicken microbiome do not correlate to changes in C. jejuni global gene expression during colonization

**Figure 2:**
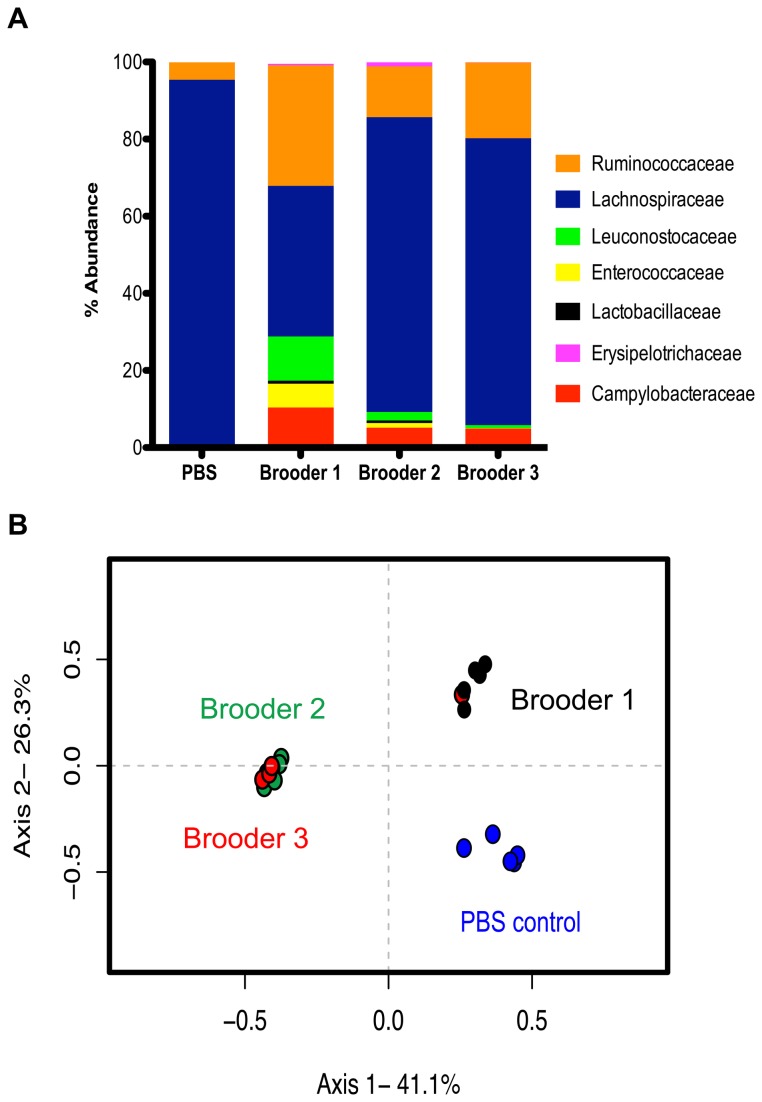
The cecal microbiota of chickens colonized with *C. jejuni*. A. Defining members of the cecal microbiota by relative rank abundance plots (>1%) at the bacterial family level. The average relative abundance for each treatment group (PBS control n=5; brooder 1 n=5; brooder 2 n=5; brooder 3 n=5) is represented in the bar plot. B. Principle coordinates analysis (PCoA) illustrating the community structure relationship between chicks from different treatment groups. This ordination was generated using a Yue and Clayton-based distance matrix representing the relative abundance of OTUs in each community at a 3% OTU definition level. The community of each chick is indicated by a colored symbol (PBS control = blue; brooder 1 = black; brooder 2 = green; brooder 3 = red). All brooder bacterial communities were significantly different from PBS controls (*p*=0.008 for brooder1 and *p*=0.007 for brooder 2 and 3; AMOVA). Brooder 1 was significantly different from Brooder 2 and 3 (*p*=0.003 and *p*=0.015, respectively; AMOVA).

To determine the relative abundance of *C. jejuni* in the chick ceca during colonization, we performed 454-pyrosequencing of the 16S rRNA gene isolated from the same *C. jejuni* colonized chicks used in RNAseq experiments, as well as PBS (mock) infected chicks. DNA based 454-pyrosequencing analysis of the V3-V5 region of the 16S rRNA gene yielded a total of 87,663 sequences, which after trimming and chimera removal by the analysis program mothur [43], yielded 48,571 sequences with minimum read length of 250 base pairs. On average, *Campylobacters* represented between 5–10% of the total number of sequences identified in the ceca of brooder 1 (10%), 2 (5%) and 3 (5%), (Figure 2A). We identified differences in the structure of the cecal microbiota at the phylum, family and OTU (Operational Taxonomic Unit) level between brooder sets and PBS control chicks (Figures 2 and S1). Previous studies investigating the cecal microbiota of pathogen free and *C. jejuni* colonized chicks showed a difference in phylotype distribution; however, no changes in the microbiota functional gene content was identified [44]. In this study, chicks colonized with *C. jejuni* had microbiome structures distinct from the PBS control group (Figure 2B). The structure of the cecal microbiota of chicks colonized with *C. jejuni* housed in brooder 1 was statistically different from chicks housed in brooders 2 and 3 (Figure 2B). Moreover, at the family level, chicks in brooder 1 showed an increase (compared to the other brooders) in the relative abundance of Ruminococcaceae, Leuconostocaceae and Enterococcaceae families and a decrease in members of the Lachnospiraceae family (Figure 2A). Brooder 1 chicks also had an increase in the relative abundance of Campylobacteraceae (~10%) compared to brooders 2 and 3 (~5%). This poses questions as to whether the indigenous cecal microbiota dictates the level of *Campylobacter* colonization, or if the level of *Campylobacter* colonization results in distinct shifts in the microbiota. Although there was a significant difference in the bacterial community structure between brooders, there was no significant difference (avg. R-value of 0.979) in the global transcriptome profiles (Figure S2, G-I), indicating that changes in the cecal microbiota, observed in this study, did not result in changes in the global gene expression of *C. jejuni*. This is in agreement with the result that changes in the microbiota of chicks colonized with *C. jejuni* did not alter functional gene content of the microbiome within the chick cecum [44]. In this study, we observed no changes in transcriptome profiles between animal replicates (Figure S2 G–I); in contrast, a previous transcriptome study of *C. jejuni*, using the rabbit illeal loop model (RIL), showed wide variances in transcript abundance between animals (R value of 0.49) [27]. The difference between the two sets of findings could be due to differences in animal models (RIL v. 1-day-old chicks), including host immune response and the host microbiota structure and function, and/or the result of differences in transcriptomic technologies (microarray v. RNAseq).

### Differential gene expression during colonization of the chicken cecum and validation of RNAseq

To analyze differential expression of transcripts between *in vivo* and *in vitro* samples we utilized the variance analysis package DESeq [45]. A substantial number of genes were significantly differentially expressed (>4-fold, p_adj_ <0.05) *in vivo* compared to both *in vitro* conditions. Differential expression profiles of chick samples compared to mid-log samples identified 149 genes that were differentially expressed; 135 transcripts showed increased abundance *in vivo* while 14 genes had decreased abundance (Figure 1B, Tables S1 and S2). Comparing DESeq of chick samples to stationary samples, 152 genes were significantly differentially expressed (>4-fold, p_adj_ <0.05); 95 transcripts with increased abundance compared to 57 with decreased abundance (Figure 1B and Tables S3 and S4). Of the genes with increased abundance *in vivo*, 29 were increased in comparison to both *in vitro* conditions (Figure 1B, Table 2). No transcripts were found to be decreased in abundance in both conditions with a >4-fold cutoff. All genes significantly differentially expressed (>4-fold, p_adj_ <0.05) during *in vivo* colonization compared to *in vitro* growth, and those differentially expressed between *in vitro* growth conditions, are listed in Tables S1-S6. Expression profiles of every gene, under all growth conditions, are listed in Table S9.

**Table 2:** Genes increased in abundance *in vivo* compared to both *in vitro* mid-log and stationary phase cultures.

	CJJ Locus Number	Gene Name / Function	Fold Change (ML)*	Fold Change (Stat)*
**Energy and Metabolism**	CJJ81176_0044	*dsbB*	4.47	8.04
	CJJ81176_0064	Cytochrome c Fmaily	10.46	20.99
	CJJ81176_0403	Sulphite Oxidase	4.52	14.00
	CJJ81176_0880	*dsbA*	5.30	5.10
**Stress Response**	CJJ81176_1387	*katA*	320.82	46.11
	CJJ81176_1574	*cgb*	12.29	4.72
**Transport**	CJJ81176_0642	*pstS*	8.00	44.54
	CJJ81176_0643	*pstA*	6.75	31.31
	CJJ81176_0644	*pstC*	7.92	5.74
	CJJ81176_0750	ABC transporter periplasmic substrate binding protein	5.36	24.95
	CJJ81176_0753	ABC transporter periplasmic substrate binding protein	6.39	11.85
	CJJ81176_0754	ABC transporter periplasmic substrate binding protein	8.69	10.82
	CJJ81176_1601	*chuA*	8.75	10.54
	CJJ81176_1602	*chuB*	6.73	5.20
	CJJ81176_1603	*chuC*	6.61	6.79
	CJJ81176_1604	*chuD*	7.91	8.19
	CJJ81176_1619	*exbB-2*	16.40	43.35
	CJJ81176_1620	*exbD*	8.59	49.24
**Other**	CJJ81176_1388	Ankyrin repeat protein	87.98	18.96
**Hypothetical**	CJJ81176_0045	Hypothetical	4.72	15.76
	CJJ81176_0063	Hypothetical	27.27	20.74
	CJJ81176_0065	Hypothetical	6.53	31.66
	CJJ81176_0522	Hypothetical	19.65	5.19
	CJJ81176_0751	Hypothetical	6.42	16.33
	CJJ81176_0752	Hypothetical	7.05	21.44
	CJJ81176_0954	Hypothetical	24.06	9.70
	CJJ81176_1062	Hypothetical	11.11	11.19
	CJJ81176_1386	Hypothetical	23.72	30.08
	CJJ81176_1746	Hypothetical	21.00	7.06

* p_adj_ < 0.05, a corrected p-value analogous to a false detection rate of < 5%.

**Figure 3:**
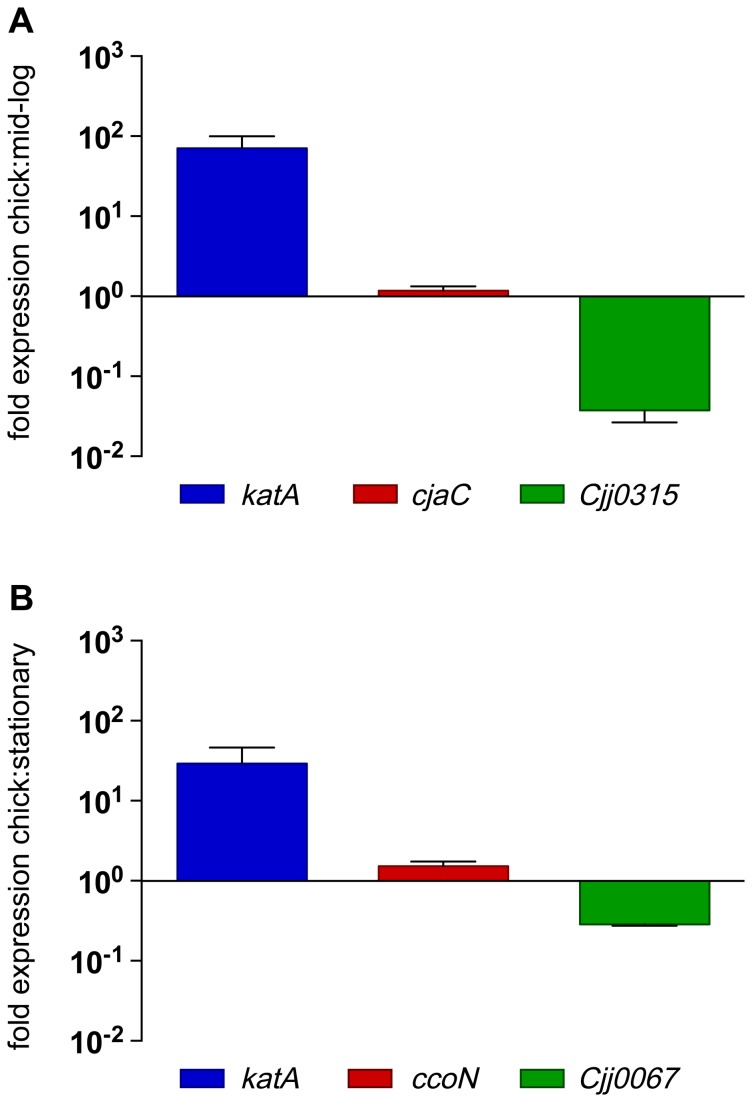
Validation of RNAseq expression analysis by qRT-PCR. Relative expression levels of genes determined by RNAseq to be differentially expressed *in vivo* compared to A. *in vitro* mid-log cultures and B. *in vitro* stationary phase cultures. Relative expression was assessed using the 2(-Delta Delta C(T)) method, error bars represent standard deviation of three independent biological replicates.

To validate expression patterns identified by RNAseq, we performed qRT-PCR on RNA isolated from *in vitro* and *in vivo* grown cultures. RNA used in qRT-PCR experiments was isolated independent from samples used in RNAseq library construction. Primers were designed to amplify genes with increased, decreased or unchanged levels of transcript abundance *in vivo* compared to *in vitro* samples (Table S8). RNAseq identified *katA* as one of the highest abundant transcripts during colonization compared to both *in vitro* growth conditions, and this increase in abundance was confirmed by qRT-PCR (Figure 3). Furthermore, genes identified as having decreased transcript abundance (*Cjj0315* and *Cjj0067*) or no changes in transcript abundance (*cjaC* and *ccoN*) during colonization, compared to *in vitro* grown cultures, were also confirmed by qRT-PCR, validating the RNAseq expression profiles (Figure 3). Because differential expression analysis (DESeq) combined and averaged sequencing results from the three biological replicates, we wanted to confirm that transcript abundance between replicates was similar. Expression profiles between each biological replicate were compared by plotting the abundance (log_2_) of each ORF between all replicates of each growth condition. Transcript abundance between replicates, of all three growth conditions, showed no statistical differences (Figure S2).

### Insights into the intestinal environment during C. jejuni colonization derived from global transcript regulatory patterns

**Figure 4:**
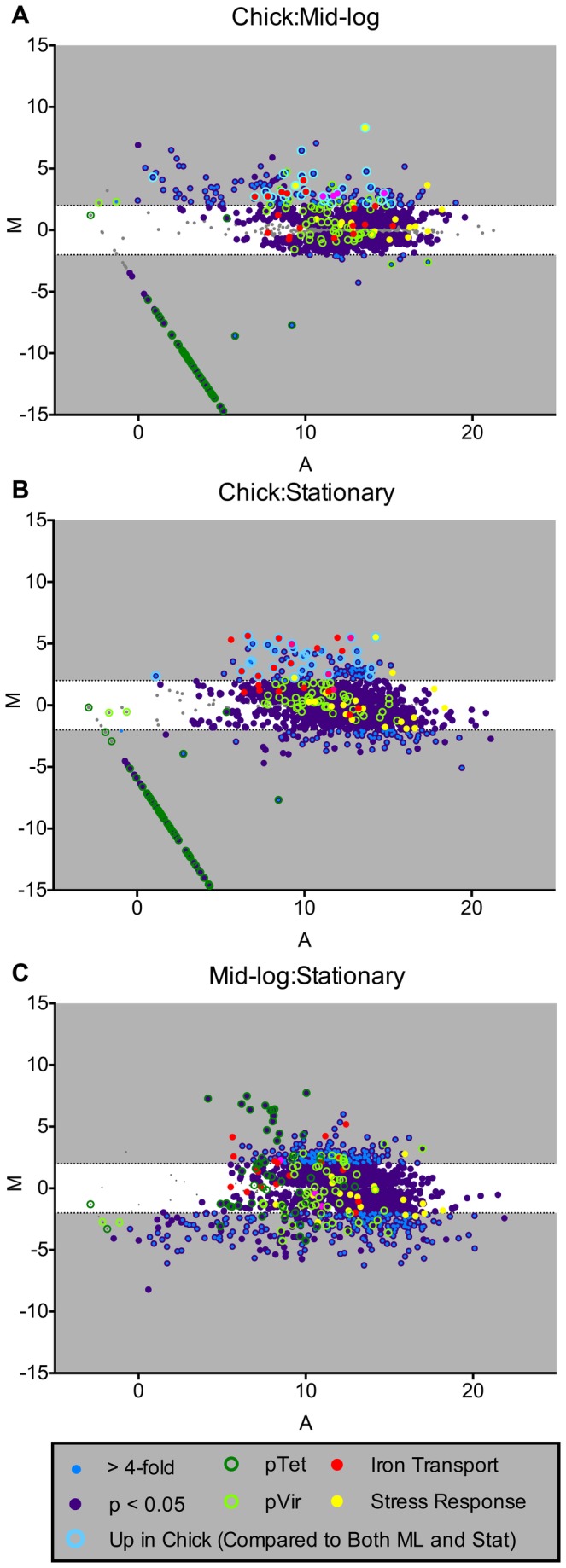
Differential gene expression (DESeq) of *C. jejuni* during *in vivo* colonization and *in vitro* growth. (A–C) The log_2_ of the ratio of abundances of each gene between the indicated conditions [M] plotted against the average log_2_ of abundance of that gene in all conditions [A]. For each plot, [M] and [A] values were generated with DESeq [45] using data from three biological replicates of each *in vivo* and *in vitro* growth condition. Genes significantly differentially expressed (>4-fold p_adj_<0.05) as well genes of specific functional groups are highlighted (see legend for functional group highlight annotation). Grey regions highlight expression differences greater-than and less-than 4-fold.

To identify global regulation patterns occurring *in vivo*, we graphed DESeq analyses on MA plots (Figure 4). MA plots represent the log_2_ of the ratio of abundances of each ORF between the indicated conditions [M], plotted against the average log_2_ abundance of that ORF in all conditions [A]. Three functional gene groups were identified as increased in abundance during colonization including iron transport, phosphate transport and oxidative and nitrosative stress responses. Iron has been consistently identified as a critical micronutrient for *C. jejuni* growth and colonization [46–50]. Increased expression of the heme transport genes *chuA* and *chuB* were previously identified in an *C. jejuni in vivo* microarray transcriptome study [18]. Utilizing RNAseq, we confirmed the increased abundance of *chuA* and *chuB* transcripts along with *chuC* and *chuD* of the heme transport operon (*chuABCD*). Additionally, we identified two other iron transporters with increased expression *in vivo*, including the outer membrane TonB-dependent energy transduction system *exbB-exbD*-*tonB* and the ferric transport system FTR1-*p19*. The increased abundance of three iron transport systems suggests that the chicken cecum is an extremely iron-limiting environment.

**Figure 5:**
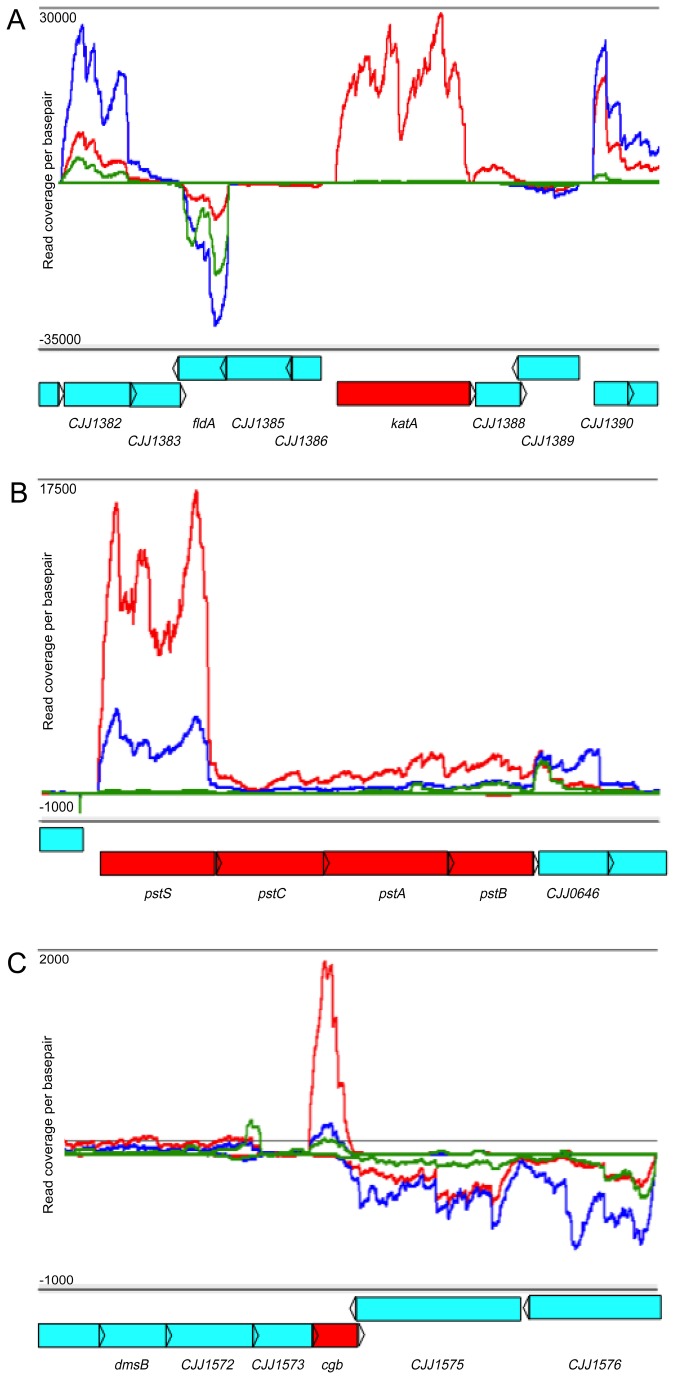
Gene expression profiles of *C. jejuni* during *in vitro* growth and *in vivo* colonization. Histograms illustrating strand-specific coverage per nucleotide across multiple loci on the *C. jejuni* chromosome. Red lines represent reads sequenced and mapped from *in vivo* chick libraries. Blue (mid-log) and green (stationary) lines represent reads sequenced and mapped from *in vitro* libraries. ORF’s are labeled below each histogram in blue. Specific loci A) *katA*, B) *pstSCAB*, C) *cgb*, which were increased in abundance during *in vivo* colonization, are highlighted in red.

Transcripts from the phosphate transport system (*pstSCAB*) were increased in abundance *in vivo* compared to both *in vitro* conditions (Figure 5B, Tables S1 and S3); however, in contrast to the iron transport systems noted above, increased expression of this locus was not observed in either previous *in vivo* microarray transcriptome study [18,27]. In addition to this ABC-type transport system, *C. jejuni* encodes a low affinity non-specific phosphate transport system *pitAB* (*Cjj1208, Cjj1209*) [51], which was not differentially expressed to any significant extent in our studies. In response to phosphate limitation, the *pst* transport system is activated by the two-component system PhoSR; *pst* is required for growth in phosphate-limited media [52] while the *pitAB* locus has yet to be characterized. A role for *pitAB* and *pstSCAB in vivo* has yet to be determined in *C. jejuni*, but increased abundance of the *pst* system *in vivo* suggests that the chick cecum is limited in phosphate and that this system could be required for colonization based on its importance *in vitro* [52].

Furthermore, increased *in vivo* transcript abundance (compared to *in vitro* growth) was observed from genes involved in oxidative and nitrosative stress responses (Figure 4, Figure 5, Tables S1 and S3). The second highest differentially expressed gene *in vivo*, *katA* (encoding catalase), is critical for the *C. jejuni* response to H_2_O_2_ and is required for colonization of the chicken cecum (Figure 5A) [53–55]. Additionally, the truncated flavohemoprotein (*cgb*), involved in the nitrosative stress response [56], was highly expressed *in vivo* (Figure 5C). The high level of expression of these two important stress response genes during *in vivo* colonization corresponds with studies demonstrating that *C. jejuni* induces a host immune response in the chicken [57–59]. Specifically, RNAseq analysis of chicken cecal tissue during colonization with *C. jejuni* showed increased expression of NOXO1, (NADPH oxidase organizer 1) which regulates respiratory burst [60]. Moreover, infection of human HCT-8 intestinal epithelial cells with *C. jejuni* activated host Nox1 and Duox2, resulting in increased H_2_O_2_ production and consequent inactivation of CjtK. CjtK, a tyrosine kinase encoded by *C. jejuni*, activates GalE, required for glycosylation and capsule formation, important factors in adherence and invasion [61]. The high level of catalase gene expression during *in vivo* growth suggests that *C. jejuni* is responding to host H_2_O_2_ production, which might be a mechanism to avoid inactivation of CjtK allowing for adherence and invasion. Furthermore, *C. jejuni* elicits a TLR response in chicks, specifically activating TLR-2, TLR-4 and TLR-21 [62]. Most importantly, TLR signaling was shown to be induced when using *C. jejuni* cell lysates compared to whole cells, proposing that bacterial lysis (evoking a hostile host environment) is required for full TLR activation [62]. Although *C. jejuni* is considered a commensal that does not induce human-like disease symptoms in chicks, it does elicit an immune response; therefore, increasing expression of stress response genes could possibly result in a mechanism for *C. jejuni* to postpone lysis, preventing a robust immune response, resulting in persistent colonization.

Overall, we identified 272 transcripts that were differentially expressed *in vivo* (Tables S1-S4). Many of the genes whose abundance increased *in vivo* contribute to cellular processes including central carbon metabolism, electron transport, biosynthetic processes and transport systems, indicative of a nutrient-restricted environment in the chick cecum compared to the rich *in vitro* growth medium MHB. We observed that *in vivo* abundance of RNA transcripts from the pTet plasmid were extremely low, with some transcripts never detected (Figure 4). This suggests either that *C. jejuni* strongly down-regulates expression of this plasmid *in vivo*, or that the plasmid is lost at a high rate during colonization. Both pTet and pVir are conjugative plasmids in *C. jejuni* 81-176 and both contain type-4 secretion system (T4SS) genes, with only genes encoded on pVir functionally characterized [63–65]. Involvement of pVir in attachment, invasion and disease during colonization of ferrets has been demonstrated [65]; however, no mechanism by which pTet might contribute to colonization has been uncovered. Genes involved in histidine biosynthesis and nitrogen metabolism (*hisH* and *hisF*) [66] were decreased *in vivo* (Table S2), suggesting that the chick cecum could be replete with histidine and/or glutamate. In *C. jejuni*, *hisH* and *hisF* have been implicated in the biosynthesis of intermediates of the flagellar glyscosylation pathway [67]; however, no role in chick colonization has been determined. Similarly, the major antigenic peptide (PEB3) was also determined to be decreased in abundance *in vivo* (Table S2). This glycoprotein is immunoreactive [68,69] suggesting that decreased expression could be a mechanism to evade host recognition leading to persistent colonization.

Comparison of RNAseq expression profiles to those of a previous *in vivo* transcriptome study using microarrays revealed many differences in expression patterns along with some similarities [18]. Most evident are the total number of differentially regulated genes between the two experiments, with microarray identifying only 68 differentially expressed genes (>2-fold), while RNAseq identified 272 differentially expressed genes, even using a higher stringency cutoff (>4-fold). Functional groups identified as differentially expressed in both experiments included components of electron transport, central carbon metabolism and transport systems. *C. jejuni* encodes a highly branched electron transport chain allowing for adaptation to specific environments [7,8,70]. *C. jejuni* is considered a microaerophile but has been shown to grow anaerobically in the presence of alternative electron acceptors including DMSO and nitrate [8]. RNAseq identified increased abundance of many transcripts associated with electron transport, including DMSO reductase, sulphite oxidase, the low-affinity oxidase (*cioAB*) and cytochrome *c* biogenesis (Tables S1-S4). Alternatively, there was decreased *in vivo* abundance of NADH: ubiquinone oxidoreductase transcripts (~2-3-fold), which encode proteins responsible for using reduced flavodixin as an electron donor instead of NADH [71], and there was no change in the abundance of the high-affinity oxidase CcoN (Figure 3B). Taken together, and as previously hypothesized, *C. jejuni* most likely encounters a limited oxygen environment in the cecum, as CioAB is a low-affinity oxidase [72], and *C. jejuni* has been shown to be able to respire off substrates such as sulphite and DMSO [8,70,73]. We did not observe an increase in expression of the nitrate reductase genes (*napAB*) as identified in the previous chick *in vivo* microarray study [18]. This difference in expression of *napAB* could be a result of growth temperature variation of *in vitro* grown cultures. The nitrate reductase locus is up-regulated at 42°C, compared to 37°C [74]. *In vitro* cultures for RNAseq experiments, reported here, were grown at 42°C in an effort to identify genes that were differentially regulated based on host colonization factors and the host nutritional environment, not differences in temperature, as the internal temperature of the chicken is 42°C. Additionally, expression differences could be due to different colonization conditions (including strain differences, inoculum load, duration of colonization and *in vitro* growth conditions). Finally, these differences could also be attributed to differences in transcriptome mapping technologies, as RNAseq affords direct quantification of transcripts and high sensitivity for low-abundance transcripts. As transcriptome studies are snapshots of gene expression, it would be interesting to determine *C. jejuni* gene expression profiles over time during the course of colonization. This would allow for a more in-depth profile of the transcriptome during initial colonization through persistent colonization.

### Identification of non-coding and anti-sense RNA species

**Figure 6:**
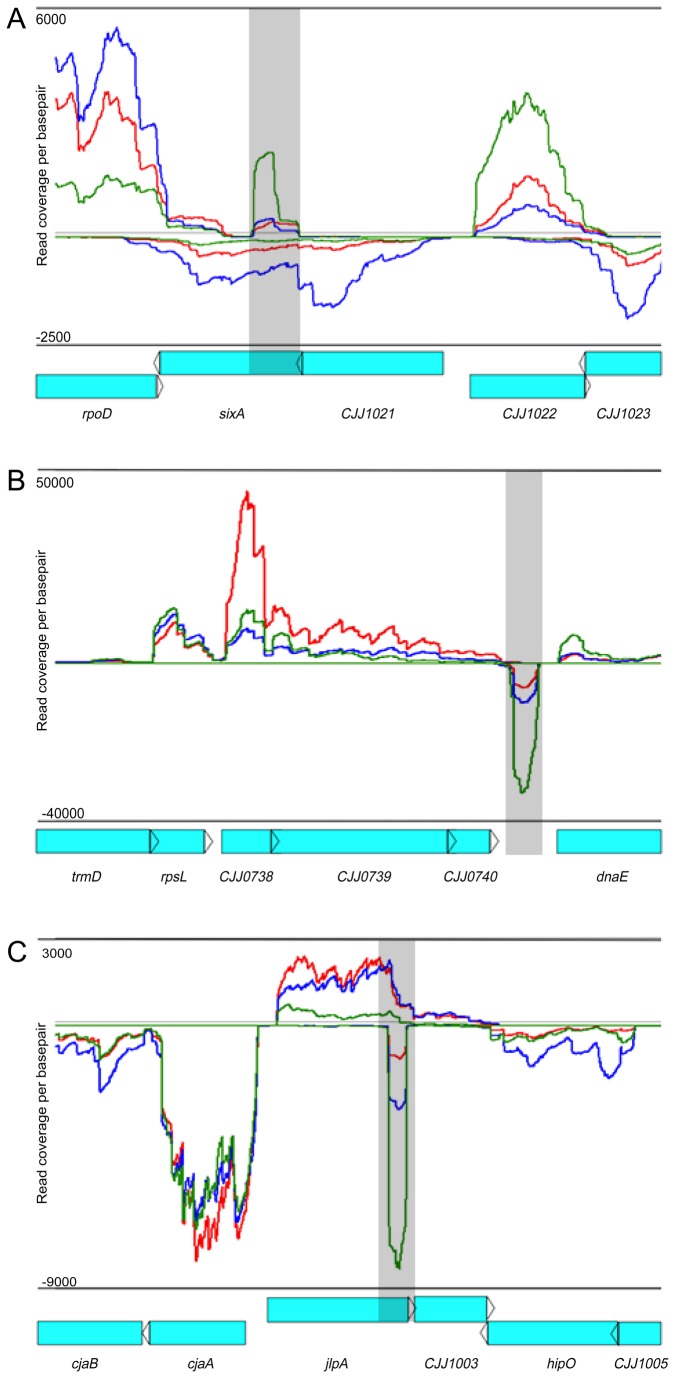
Expression profiles of non-coding RNA species during *in vitro* growth and *in vivo* colonization. Histograms illustrating strand-specific coverage per nucleotide across multiple loci identifying non-coding RNA species. Red lines represent reads sequenced and mapped from *in vivo* chick libraries. Blue (mid-log) and green (stationary) lines represent reads sequenced and mapped from *in vitro* libraries. ORF’s are labeled below each histogram in blue. Specific ncRNAs, A. anti-sense nc4, B. intergenic nc18, C. anti-sense nc21, are highlighted by grey boxes.

We next mined our RNAseq data for non-coding RNAs and identified 51 putative ncRNA candidates on the chromosome (Table 3) and on plasmid pTet (Table S7), including transcripts derived from intergenic regions and anti-sense to open reading frames (Figure 6). Our analysis identified nearly all the *C. jejuni* ncRNAs annotated in the Rfam database [75], including the thiamin pyrophosphate (TPP) riboswitch, the bacterial signal recognition particle (SRP), tmRNA and the RNA component of RNase P [9,76]. Similarly, we detected expression of 17 of the 25 ncRNAs identified in previous *C. jejuni* RNAseq studies [29,30]. In several cases, expression of ncRNAs previously identified in *C. jejuni* strain NCTC11168 were not detected in our analysis of strain 81-176, despite the conservation of these loci in both strains, an observation consistent with recent studies in *Campylobacter* sp. [30] and *Listeria* sp. [77] showing that expression of conserved ncRNAs may diverge significantly even among strains of the same species. Additionally, several ncRNAs previously identified in *C. jejuni* 81-176 [30] were not identified in our study, likely reflecting differences in growth temperatures, RNA library construction protocols, and/or ncRNA prediction algorithms.

**Table 3:** Non-coding RNAs identified by RNAseq and their differential expression under different growth conditions.

ncRNA ID	Chromosome Location	DESeq Chick:ML	DESeq Chick:Stat	DESeq ML:Stat
**nc 1**	100050.100258	4.10	0.18	0.05
**nc 2**	104909.105040	5.89	0.44	0.08
**nc 3**	913140.913278	1.10	0.07	0.07
**nc 4**	940044.940212	2.87	0.17	0.06
**nc 5**	1032051.1032145	17.74	0.44	0.03
**nc 6**	1190593.1190723	1.58	0.21	0.14
**nc 7**	1278300.1278473	0.81	0.15	0.19
**nc 8**	1536244.1536387	15.56	0.32	0.02
**nc 9**	1566220.1566328	2.42	0.37	0.16
**nc 10**	77586.77846	0.65	0.84	1.34
**nc 11**	93760.93891	1.00	0.19	0.20
**nc 12**	227132.227266	20.33	0.71	0.04
**nc 13**	176680.176901	5.11	0.31	0.06
**nc 14**	250947.251049	5.28	0.79	0.16
**nc 15**	506475.506785	11.66	0.21	0.02
**nc 16**	578796.579113	1.85	0.31	0.17
**nc 17**	622442.622594	6.59	0.15	0.02
**nc 18**	667317.667510	1.35	0.13	0.10
**nc 19**	874073.874227	0.58	0.04	0.07
**nc 20**	878326.878438	6.90	0.38	0.06
**nc 21**	924301.924469	0.89	0.09	0.10
**nc 22**	1036626.1036898	1.85	0.26	0.15
**nc 23**	1075868.1076095	3.30	0.56	0.18
**nc 24**	361854.361960	1.47	0.10	0.07
**nc 25**	1248138.1248316	2.10	0.23	0.11
**nc 26**	1502121.1502247	2.65	0.08	0.03
**nc 27**	1551155.1551721	10.12	1.32	0.14
**nc 28**	457707.457860	10.98	3.34	0.32
**nc 29**	459841.460022	1.06	0.06	0.06
**nc 30**	473652.473826	19.98	0.59	0.03
**nc 31**	789226.789789	1.17	0.39	0.35
**nc 32**	878386.878507	2.52	0.07	0.03
**nc 33**	910988.911157	59.73	2.89	0.05

**Figure 7:**
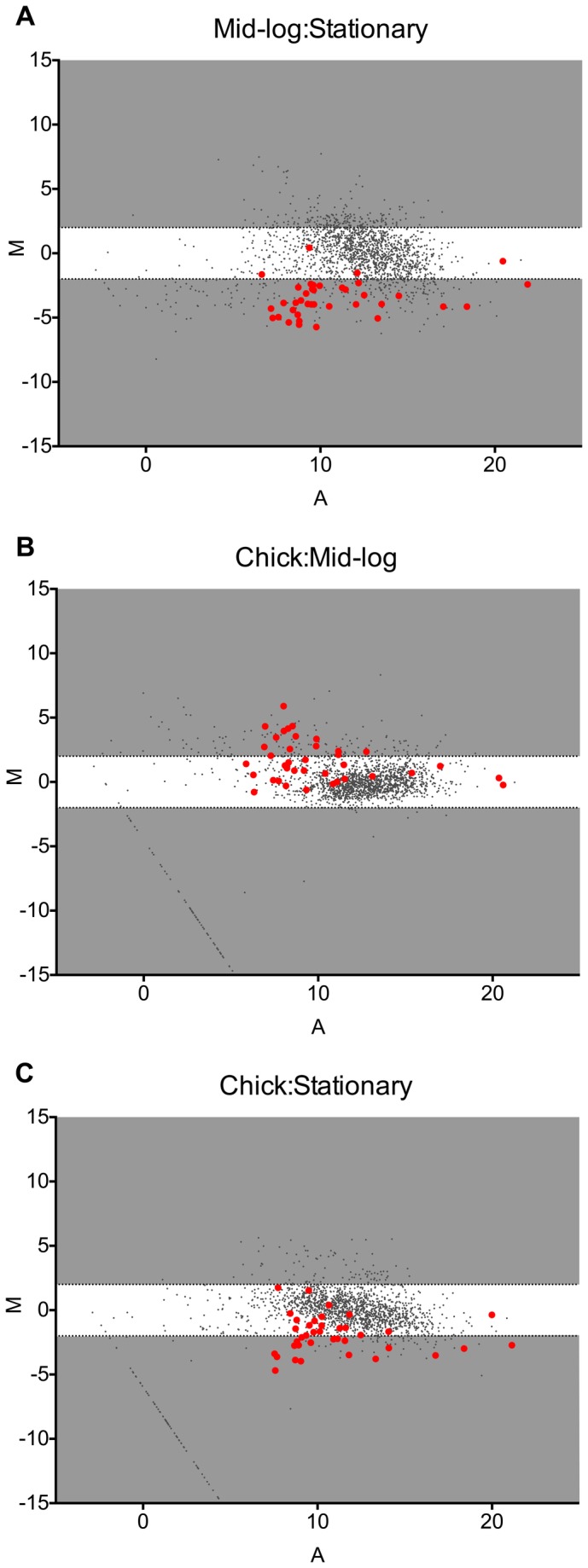
Differential expression of putative non-coding RNA species identified by RNAseq and Rfam analyses. (A–C) The log_2_ of the ratio of abundances of each gene between the indicated conditions [M] plotted against the average log_2_ of abundance of that gene in all conditions [A]. For each plot, [M] and [A] values were based on data from three biological replicates from each *in vivo* and *in vitro* growth condition. ORFs are marked as dark grey circles. Non-coding RNAs are highlighted in red. Grey regions highlight expression differences greater-than and less-than 4-fold.

The abundance of most of these candidate ncRNAs increased during stationary phase compared to mid-log phase (Figure 7A), suggesting they are likely induced in response to increased stress and/or nutrient limitation. The abundance of these putative ncRNAs was also induced *in vivo* compared to mid-log cultures, though not as much as in stationary phase (Figure 7B and 7C), again suggesting their expression is likely regulated by similar stimuli and conditions encountered by *C. jejuni* during its transition from log to stationary growth in culture.

To investigate possible regulatory mechanisms of the newly identified ncRNAs we utilized the sRNA target prediction program TargetRNA [78]. Non-coding RNAs are a unique class of regulators that can regulate gene expression by different mechanisms, including altering mRNA interactions with the ribosome [79] and modulating mRNA stability [80–83]. Additionally, ncRNAs can act as cis-regulatory elements or as trans-regulatory elements [84]. We chose to focus on the intergenic ncRNAs for TargetRNA analysis, as these are more likely to act as trans-regulatory elements and potentially have multiple targets. TargetRNA identified at least 4 targets (p-value < 0.01) for eight intergenic encoded ncRNAs (Table 4). The pattern of abundance of many of these targets was the same or opposite of their cognate ncRNAs among the conditions tested (Table 4), suggesting their stability may be regulated by the ncRNA and lending further credence to the TargetRNA predictions. Those targets whose expression patterns were not well correlated with their cognate ncRNA may represent false predictions or correspond to ncRNA: mRNA interactions that do not significantly alter message stability. Further characterization of these putative ncRNAs and target mRNAs will likely yield key insights into *C. jejuni* regulatory mechanisms and pathways.

**Table 4:** mRNA targets of ncRNAs analyzed by TargetRNA and their differential expression in different growth conditions.

ncRNA ID	Gene Name / locus number	TargetRNA Score	DESeq of mRNA Target				
			**Chick:ML**		**Chick:Stat**	**ML:Stat**	
**nc3**	CJJ81176_0481	-84	3.01	1.02			-2.82
	*mviN*	-80	1.04	-1.91			-1.89
	CJJ81176_0193	-77	-2.08	1.81			3.96
	CJJ81176_0024	-77	2.11	-6.92			-13.98
**nc5**	CJJ81176_1304	-96	1.16	-1.62			-1.79
	CJJ81176_0289	-93	1.40	1.01			1.08
	CJJ81176_1389	-81	1.37	1.35			1.04
	*porA*	-79	-1.02	-1.55			-1.44
**nc6**	CJJ81176_0565	-112	1.40	-1.33			-1.77
	*tuf*	-107	2.31	-1.46			-3.22
	*rpsG*	-105	1.61	1.64			1.06
	*flgB*	-102	2.63	-4.63			-11.63
**nc9**	*chuA*	-91	8.75	10.54			1.26
	*pgk*	-86	-1.15	-1.19			1.01
	*neuB1*	-84	-1.31	1.68			2.31
	*fliN*	-79	1.09	-2.57			-2.67
**nc14**	*argC*	-79	-1.65	-1.22			1.42
	*mutY*	-75	-1.75	2.02			3.71
	CJJ81176_0579	-74	-1.78	-2.41			-1.29
	CJJ81176_0213	-68	-2.21	1.63			3.78
**nc18**	CJJ81176_1193	-93	1.23	-1.84			-2.17
	CJJ81176_0481	-84	3.01	1.02			-2.82
	*secF*	-71	-1.71	1.42			2.53
	CJJ81176_0973	-70	-1.79	-1.53			1.22
**nc29**	CJJ81176_0193	-96	1.16	1.81			3.96
	*nuoM*	-87	-2.07	2.45			5.31
	CJJ81176_0481	-84	3.01	1.01			-2.82
	*mviN*	-80	1.04	-1.90			-1.90
**nc32**	CJJ81176_1746	-85	21.01	7.06			-2.85
	*mobB*	-84	1.64	-2.03			-3.19
	CJJ81176_1370	-83	-1.06	-1.32			-1.19
	*nspC*	-80	-1.97	-1.74			1.19
**nc33**	*cutE*	-93	2.38	4.59			2.02
	*tlyA*	-84	-1.57	1.89			3.11
	CJJ81176_0076	-79	1.27	2.42			1.98
	*napL*	-79	-2.08	-1.72			1.27

The small size of its genome, its lack of an Hfq homologue, and the relatively limited success of genomics-based approaches for identifying ncRNAs in *C. jejuni* [85] have led to speculations that *C. jejuni* does not encode the sizeable arsenal of ncRNAs found in many other bacteria. However, the discovery of numerous putative *C. jejuni* ncRNAs in this study, and in other recent RNAseq-based studies, suggests that *C. jejuni* does indeed possess a robust RNA-mediated regulation system and provides insights into the mechanisms by which this organism, which encodes so few canonical transcription factors, is able to effectively adapt to rapidly changing environmental conditions and niches.

### The *Campylobacter jejuni* RNAseq transcriptome browser

The RNAseq data generated during the course of this study contains a wealth of information that will be a valuable resource to the numerous researchers studying *C. jejuni*. To make these data readily accessible to this community, we have generated coverage plots for each RNAseq dataset that can be visualized in the GenomeView browser [86] using the following link:

http://www.broadinstitute.org/software/genomeview/supplemental/CjVJD13/. This browser allows visualization of expression profiles of all annotated ORFs (under all growth conditions), as well as non-coding RNA species; including 5’-UTRs, anti-sense transcripts and intergenic ncRNAs.

## Summary

This is the first report of the complete transcriptome of *C. jejuni* during colonization of the chicken cecum using RNAseq. Through our strand specific library preparation, we identified 51 probable non-coding RNA species, 29 of which were not previously annotated. We also determined the expression profile of *C. jejuni* during colonization, identifying 272 genes that are significantly differentially expressed *in vivo*. Additionally, we characterized the microbiota of the chick cecum during *C. jejuni* colonization. It was shown that *C. jejuni* represents ~5-10% of the bacterial population and that structural differences in microbiota profiles do not result in changes in the global *C. jejuni* transcriptome. *C. jejuni* must rapidly regulate gene expression in response to diverse environmental conditions; however, the regulatory mechanisms underpinning these responses are poorly understood. This work provides key insights into the genes and functions involved in the transition of *C. jejuni* from logarithmic to stationary growth and during its adaption to the chicken cecal environment, as well as revealing a number of putative regulatory ncRNAs that may play key roles in these adaptations.

## Materials and Methods

### Bacterial strains and growth conditions

*C. jejuni* strain DRH212 (an 81-176 streptomycin resistant derivative [87]) was grown on Mueller Hinton agar (BD, Sparks, MD) supplemented with 10% sheep blood (BA) or in Mueller Hinton Broth (MHB) (BD, Sparks, MD). *C. jejuni* was cultured at 42°C microaerobically in a tri-gas incubator, constantly maintained at 5% O_2_, 10% CO_2_, balanced with N_2_. Liquid cultures of *C. jejuni* were grown in MHB to mid-log or stationary phase (as monitored by optical density Ab_600_).

### Chicken Colonization

Animal work carried out in this study followed the recommendations of the National Institutes of Health in the Guide for the Care and Use of Laboratory Animals. This protocol was approved by the University Committee on Use and Care of Animals at the University of Michigan (Protocol 10462-1). All efforts were made to minimize suffering of animals throughout the course of the experiment.

One-day-old chicks were inoculated orally with 1x10^3^ CFU of *C. jejuni* in PBS. Cells used in the inoculum were grown on BA for ~16 h, washed and resuspended in PBS to 10^4^ CFU/mL. Chicks were inoculated with 100 µl of cell suspension via oral gavage. Seven days post inoculation, chicks were sacrificed and cecal contents were removed. For RNA isolation, cecal contents were immediately suspended in 1 mL of RNAlater (Qiagen, Valencia CA) and frozen at -80°C. For colonization load and DNA extraction for 454-pyrosequencing, a subsample of cecal contents was removed and diluted in PBS. Colonization load was determined by serially diluting cecal contents and plating on *Campylobacter* selective media (BA supplemented with Vancomycin [40 µg/ml], Cefoparazone [40 µg/ml], Trimethoprim [10 µg/ml] and Cycloheximide [100 µg/ml]). Plates were incubated at 42°C under microaerobic conditions until countable colonies appeared (~2-3 days). CFU counts were standardized to gram wet weight of cecal content.

### RNA isolation

*In vitro* RNA was isolated from *C. jejuni* grown in MHB under microaerobic conditions to mid-log and stationary phase (as monitored by optical density Ab_600_). An equal volume of RNA stop solution (95% EtOH, 5% phenol) was added to the cultures immediately after removal from microaerobic conditions and cells were harvested by centrifugation at 4°C. RNA was extracted with TRIzol® (Invitrogen, Grand Island, NY) as per manufacturer’s instructions. Contaminating DNA was removed with two treatments of TURBO™ DNase (Invitrogen) per manufacturer’s instructions. DNase treated RNA was cleaned with RNA clean and concentrate 25 columns after each DNase treatment step (Zymo Research, Irvine, CA). Conformation of DNA removal was assessed by PCR with three sets of primers ranging in amplification products of 100, 150, 250 bp and the Qubit dsDNA high sensitivity assay kit (Life Technologies) (data not shown).

For *in vivo* RNA isolation, cecal contents were harvested from individual birds and immediately submerged in 1 ml of RNAlater/cecum. *C. jejuni* was enriched from cecal contents as performed by Jerome et al. in an effort to deplete contaminating eukaryotic cells and cecal debris [88]. After *C. jejuni* enrichment, RNA was extracted with TRIzol® and contaminating DNA was removed with TURBO™ DNase as described previously. Purified DNA-free RNA from 5 birds (housed in the same brooder) was pooled to create enough starting DNA-free RNA for Illumina cDNA library construction.

### Illumina Library Construction

Illumina cDNA libraries were constructed in a similar manner to Mandlik et al. [31]. Five micrograms of RNA was depleted of 16S and 23S rRNA species using the Gram Negative Ribo-Zero™ rRNA Removal Kit (Epicentre, Madison, WI). Removal of contaminating RNA species was assessed with the Agilent Bioanalyzer RNA 6000 nano chip (Agilent Technologies, Santa Clara, CA). Depleted mRNA was fragmented into 100-500 bp species with fragmentation buffer from the GeneChip® clean up module kit (Affymetrix, Santa Clara, CA). First-strand cDNA was synthesized with random hexamers, Actinomycin D and SuperScript III (Life Technologies, Grand Island, NY). Second strand cDNA was synthesized with dUTP replacing dTTP as described by Levin et al [89]. Double stranded cDNA ends were repaired and adenylated as described in the Illumina Truseq™ RNA sample preparation low throughput (LT) protocol (Illumina, San Diego, CA). Bar-coded Illumina adapters were ligated to the ends of the cDNA libraries and adapter-cDNA libraries were treated with Uracil-N-glycosylase (UNG) for 15 min at 37°C, followed by 95°C for 5 min. UNG-treaded cDNA was enriched by low-cycle PCR (8-cylces) with Illumina adapter specific primers. Final cDNA libraries were cleaned with two treatments of AMPureXP beads (Beckman Coulter, Brea, CA), and sequenced using 50 bp paired-end reads on an Illumina HiSeq2000 platform at the University of Michigan Sequencing Core.

### Quantitative reverse transcriptase polymerase chain reaction

PCR primers used in this study are listed in Table S8 and were used to amplify nucleotide fragments of genes of interest between 100 and 150 bp. Isolation of RNA from *in vitro* and *in vivo* cultures (independent from RNAseq library preparations) was performed as mentioned previously. qRT-PCR was performed by using the Quantitect Sybr, Green RT-PCR kit (Qiagen, Valencia, CA) as by Taveirne et al. [17] and analyzed using the 2(-Delta Delta C(T)) method [90].

### RNAseq analysis

Reads were aligned to the *C. jejuni* chromosome, pTet, and pVir sequence files (RefSeq NC_008787, NC_008770, and NC_008790) using BWA [91] version 5.9. Gene annotations were obtained from RefSeq and Rfam [75]. The overall fragment coverage of genomic regions corresponding to features such as ORFs and rRNAs was conducted as described [31]. Differential expression analysis was conducted using DESeq [45].

### Chick ceca microbiota analysis

DNA extraction was done on untreated chick cecal content, in PBS, using Roche MagNA Pure Compact system according to the Roche MagNA Pure Nucleic Acid Isolation Kit I protocol instructions (Roche, Madison, WI). Amplification of the V3-V5 region of the 16S rRNA gene was accomplished using the Broad HMP protocol (HMP MDG Default Protocol v 4.2). Briefly, extracted DNA was used to construct DNA libraries targeting the V3-V5 hypervariable regions of the 16S rRNA gene using primers 357F (5’-CCTACGGGAGGCAGCAG-3’) with a B adaptor (5’-CCTATCCCCTGTGTGCCTTGGCAGTCTCAG-3’) and 926R (5’-CCGTCAATTCMTTTRAGT-3’) with a unique bar code to identify the target and an A adaptor (5’-CCATCTCATCCCTGCGTGTCTCCGACTCAG-3’). A PCR reaction mixture of 1X AccuPrime PCR Buffer II (Invitrogen), 0.15 µl AccuPrime Taq DNA Polymerase High Fidelity, 0.2 µM Primer B, 0.2 µM uniquely bar-coded Primer A was used. DNA was amplified using the following conditions: 95°C for 2 min, 30 cycles of 95°C for 20 sec, 50°C for 30 sec and 72°C for 5 min. Amplicons were cleaned with the Agencourt AMPure XP and quantified using the Quant-it PicoGreen dsDNA kit (Invitrogen). Samples were pooled in equal proportion, cleaned again with AMPureXP, and sequenced on the Roche 454 GS Junior Titanium platform according to the manufacturer’s specifications. Analysis of 454 Pyrosequencing data was done using mothur (version 1.30.2) [43]. Mothur was used to group or assign bacterial 16S rRNA gene sequences into Operational Taxonomic Units (OTUs) using a 3% species level definition. Classifications were determined by comparing sequences to the Ribosomal Database Project [92]. Classified OTUs were used to determine the relative abundance of bacterial phyla and family in each sample. Principal coordinates analysis (PCoA) was used to assess community similarity among all samples and was calculated based on Yue and Clayton-based distance matrix [93]. An AMOVA (analysis of molecular variance) was performed to determine statistical differences between the community structures of each treatment group. Heatmaps of microbiome data (OTUs) were made using the *heatmap* command in R [94]. The *heatmap* command re-orders the data in the rows and the columns separately, so that similar data are grouped together by hierarchical clustering, as shown by dendrograms for the rows and the columns. The Euclidean distance was used in the clustering [95].

## Supporting Information

Figure S1 **Structural changes to the chicken cecal microbiota.** A heatmap of the top 25 (>1%) operational taxonomic units (OTUs) found in the chick cecal microbiome shows differences between the four-treatment groups (PBS control n=5; brooder 1 n=5; brooder 2 n=5; brooder 3 n=5). The distance between treatment groups was measured by a dendrogram in R statistical program. The PBS controls (blue bars) group very distinctly from chicks colonized with *C. jejuni* in brooder 1 (black bars) and brooders 2 and 3 (green and red bars). Bacterial phylum, family and OTU number are listed on the heatmap. The heatmap scale ranges from 0 to 100% relative OTU abundance.
(TIF)

Figure S2 **Comparison of gene expression between biological replicates used in DESeq analysis.** Scatter plots of the log_2_ (FPKMO) abundance of open reading frames obtained from RNAseq libraries created from *in vitro* mid-log samples (A–C), *in vitro* stationary phase samples (D–F) and *in vivo* chick samples (G–I). R^2^ (coefficient of determination) was determined by linear regression, represented by the sold line.
(TIF)

Table S1 **Genes increased in abundance *in vivo* compared to *in vitro* mid-log phase cultures.** Listed are genes with increased abundance during *in vivo* colonization compared to *in vitro* mid-exponential phase broth grown cultures, as determined by DESeq analysis (materials and methods). Only genes significantly differentially regulated (>4-fold difference in abundance, p_adj_<0.05) are listed. p_adj_<0.05, is a corrected p-value analogous to a false detection rate of < 5%. Genes are grouped by functional classification and by their *C. jejuni* 81-176 locus numbers and gene name or function.
(DOCX)

Table S2 **Genes decreased in abundance *in vivo* compared to *in vitro* mid-log phase cultures.** Listed are genes with decreased abundance during *in vivo* colonization compared to *in vitro* mid-exponential phase broth grown cultures, as determined by DESeq analysis (materials and methods). Only genes significantly differentially regulated (>4-fold difference in abundance, p_adj_<0.05) are listed. p_adj_<0.05, is a corrected p-value analogous to a false detection rate of < 5%. Genes are grouped by functional classification and by their *C. jejuni* 81-176 locus numbers and gene name or function.
(DOCX)

Table S3 **Genes increased in abundance *in vivo* compared to *in vitro* stationary phase cultures.** Listed are genes with increased abundance during *in vivo* colonization compared to *in vitro* stationary phase broth grown cultures, as determined by DESeq analysis (materials and methods). Only genes significantly differentially regulated (>4-fold difference in abundance, p_adj_<0.05) are listed. p_adj_<0.05, is a corrected p-value analogous to a false detection rate of < 5%. Genes are grouped by functional classification and by their *C. jejuni* 81-176 locus numbers and gene name or function.
(DOCX)

Table S4 **Genes decreased in abundance *in vivo* compared to *in vitro* stationary phase cultures.** Listed are genes with decreased abundance during *in vivo* colonization compared to *in vitro* stationary phase broth grown cultures, as determined by DESeq analysis (materials and methods). Only genes significantly differentially regulated (>4-fold difference in abundance, p_adj_<0.05) are listed. p_adj_<0.05, is a corrected p-value analogous to a false detection rate of < 5%. Genes are grouped by functional classification and by their *C. jejuni* 81-176 locus numbers and gene name or function.
(DOCX)

Table S5 **Genes increased in abundance *in vitro* mid-log compared to *in vitro* stationary phase cultures.** Listed are genes with increased abundance during *in vitro* mid-exponential phase broth grown cultures compared to *in vitro* stationary phase broth grown cultures, as determined by DESeq analysis (materials and methods). Only genes significantly differentially regulated (>4-fold difference in abundance, p_adj_<0.05) are listed. p_adj_<0.05, is a corrected p-value analogous to a false detection rate of < 5%. Genes are grouped by functional classification and by their *C. jejuni* 81-176 locus numbers and gene name or function.
(DOCX)

Table S6 **Genes decreased in abundance *in vitro* mid-log compared to *in vitro* stationary phase cultures.** Listed are genes with decreased abundance during *in vitro* mid-exponential phase broth grown cultures compared to *in vitro* stationary phase broth grown cultures, as determined by DESeq analysis (materials and methods). Only genes significantly differentially regulated (>4-fold difference in abundance, p_adj_<0.05) are listed. p_adj_<0.05, is a corrected p-value analogous to a false detection rate of < 5%. Genes are grouped by functional classification and by their *C. jejuni* 81-176 locus numbers and gene name or function.
(DOCX)

Table S7 **Non-coding RNAs that align to pTet.** Location of non-coding RNA species identified by RNAseq that map to the pTET plasmid.
(DOCX)

Table S8 **Primers used in qRT-PCR experiments.** Listed are the primer sets, along with their sequence, used in qRT-PCR experiments. Each primer set is annotated by which gene it will amplify.
(DOCX)

Table S9 **Results of DEseq analysis of RNAseq data for all annotated *C. jejuni* ORFs.** Each annotated gene in the *C. jejuni* genome, and two plasmids, is listed in locus number order. DESeq analysis for each gene under each growth condition comparison is given, including the base mean reads for each growth condition and the fold change (and log_2_ fold change) between each condition. p_adj_ is the adjusted P values for each gene and resVarA/B are the variance values within each set of biological replicates.
(XLS)
