# Supplementary material for: The Complete Campylobacter jejuni Transcriptome during Colonization of a Natural Host Determined by RNAseq
Source: PLoS One. 2013 Aug 21;8(8):e73586. doi: 10.1371/journal.pone.0073586 (PMC3749233; doi:10.1371/journal.pone.0073586)
Supplement: Table S1 — Genes increased in abundance in vivo compared to in vitro mid-log phase cultures. Listed are genes with increased abundance during in vivo colonization compared to in vitro mid-exponential phase broth grown cultures, as determined by DESeq analysis (materials and methods). Only genes significantly differentially regulated (>4-fold difference in abundance, padj<0.05) are listed. padj<0.05, is a corrected p-value analogous to a false detection rate of < 5%. Genes are grouped by functional classification and by their C. jejuni 81-176 locus numbers and gene name or function. (DOCX) [file pone.0073586.s003.docx]

Table S1. Genes increased in abundance *in vivo* compared to *in vitro* mid-log phase cultures.

| Function Classification | CJJ Locus Number | Gene Name / Function | Fold Change* |
| --- | --- | --- | --- |
| Biosynthetic Processes | CJJ81176_0739 | 3-deoxy-7-phosphoheptulonate synthase | 5.06 |
|  | CJJ81176_0722 | *glnA* | 6.39 |
|  | CJJ81176_1216 | *metE* | 7.22 |
|  | CJJ81176_1217 | *metF* | 4.31 |
|  | CJJ81176_1198 | Phospholipid synthase | 11.93 |
|  | CJJ81176_0370 | *trpD* | 4.98 |
|  | CJJ81176_0369 | *trpE* | 7.51 |
| Chaperone Protein Folding | CJJ81176_0537 | *clpB* | 35.86 |
|  | CJJ81176_1243 | *dnaJ* | 5.39 |
|  | CJJ81176_0775 | *dnaK* | 8.91 |
|  | CJJ81176_1234 | *groEL* | 4.68 |
|  | CJJ81176_1233 | *groES* | 7.06 |
|  | CJJ81176_0774 | *grpE* | 9.22 |
| Energy and Metabolism | CJJ81176_1214 | 2OG-Fe(II) oxidoreductase | 19.65 |
|  | CJJ81176_0066 | Cytochrome c biogenesis | 5.12 |
|  | CJJ81176_0064 | Cytochrome c family | 10.46 |
|  | CJJ81176_1571 | *dmsB* | 4.76 |
|  | CJJ81176_0880 | *dsbA* | 5.30 |
|  | CJJ81176_0044 | *dsbB* | 4.47 |
|  | CJJ81176_0439 | Putative oxidoreductase | 4.73 |
|  | CJJ81176_0183 | *trx* | 4.33 |
|  | CJJ81176_0403 | Sulphite oxidase | 4.52 |
| Motility | CJJ81176_0572 | *flaG* | 4.20 |
|  | CJJ81176_0271 | MCP | 4.92 |
| Stress Response | CJJ81176_0356 | *ahpC* | 12.61 |
|  | CJJ81176_1574 | Truncated Flavohemoprotein | 12.29 |
|  | CJJ81176_1387 | *katA* | 320.82 |
|  | CJJ81176_0182 | *trxB* | 6.53 |
| Transcriptional Regulator | CJJ81176_0344 | FUR family transcriptional regulator | 4.76 |
|  | CJJ81176_1244 | MerR family transcriptional regulator | 5.03 |
|  | CJJ81176_0597 | Putative transcriptional regulator | 6.22 |
|  | CJJ81176_1542 | Transcriptional Regulator | 7.77 |
| Transport | CJJ81176_0087 | 2-oxoglutarate/malate translocator | 9.64 |
|  | CJJ81176_0755 | ABC transporter | 7.46 |
|  | CJJ81176_0750 | ABC transporter periplasmic substrate binding protein | 5.36 |
|  | CJJ81176_0754 | ABC transporter permease | 8.69 |
|  | CJJ81176_0753 | ABC transporter permease | 6.39 |
|  | CJJ81176_0086 | Anion transporter | 6.20 |
|  | CJJ81176_1601 | *chuA* | 8.75 |
|  | CJJ81176_1602 | *chuB* | 6.73 |
|  | CJJ81176_1603 | *chuC* | 6.61 |
|  | CJJ81176_1604 | *chuD* | 7.91 |
|  | CJJ81176_0683 | Di-/tripeptide transporter | 4.65 |
|  | CJJ81176_1619 | *exbB2* | 16.40 |
|  | CJJ81176_1620 | *exbD* | 8.59 |
|  | CJJ81176_0644 | *pstA* | 7.92 |
|  | CJJ81176_0645 | *pstB* | 6.93 |
|  | CJJ81176_0643 | *pstC* | 6.75 |
|  | CJJ81176_0642 | *pstS* | 8.00 |
|  | CJJ81176_1060 | Putative transmembrane transporter | 8.99 |
|  | CJJ81176_0088 | Sodium transporter | 17.10 |
|  | CJJ81176_0085 | Sodium transporter | 11.17 |
|  | CJJ81176_0738 | Transthyretin like protein | 9.03 |
| Other | CJJ81176_0013 | Acetyltransferase | 4.77 |
|  | CJJ81176_1388 | Ankyrin repeat protein | 87.98 |
|  | CJJ81176_0760 | Hemagglutination domain protein | 13.45 |
|  | CJJ81176_0108 | Hemerythrin iron protein | 19.58 |
|  | CJJ81176_0773 | *hrcA* | 8.90 |
|  | CJJ81176_0010 | LOS Biosysnthesis | 14.33 |
|  | CJJ81176_1215 | NLPA family lipoprotein | 10.07 |
|  | CJJ81176_0793 | NLPA family lipoprotein | 4.15 |
|  | CJJ81176_0595 | Putative ATP | 4.42 |
|  | CJJ81176_1492 | Putative inner membrane protein | 7.54 |
|  | CJJ81176_0990 | Putative periplasmic protein | 56.25 |
|  | CJJ81176_0524 | Putative periplasmic protein | 37.22 |
|  | CJJ81176_0991 | Putative periplasmic protein | 36.50 |
|  | CJJ81176_1741 | Putative periplasmic protein | 11.47 |
|  | CJJ81176_0527 | Putative periplasmic protein | 10.65 |
|  | CJJ81176_0525 | Putative periplasmic protein | 6.39 |
|  | CJJ81176_1623 | Putative periplasmic protein | 6.19 |
|  | CJJ81176_0526 | Putative periplasmic protein | 5.88 |
|  | CJJ81176_pVir0025 | Para protein | 5.40 |
|  | CJJ81176_pVir0038 | pTet39 | 4.50 |
| Hypothetical | CJJ81176_0758 | Hypothetical | 133.49 |
|  | CJJ81176_0523 | Hypothetical | 90.60 |
|  | CJJ81176_0759 | Hypothetical | 36.63 |
|  | CJJ81176_0445 | Hypothetical | 33.29 |
|  | CJJ81176_0063 | Hypothetical | 27.27 |
|  | CJJ81176_1742 | Hypothetical | 25.17 |
|  | CJJ81176_0954 | Hypothetical | 24.06 |
|  | CJJ81176_1386 | Hypothetical | 23.72 |
|  | CJJ81176_0109 | Hypothetical | 22.06 |
|  | CJJ81176_1746 | Hypothetical | 21.00 |
|  | CJJ81176_0522 | Hypothetical | 19.65 |
|  | CJJ81176_0952 | Hypothetical | 19.42 |
|  | CJJ81176_0949 | Hypothetical | 15.87 |
|  | CJJ81176_0232 | Hypothetical | 15.19 |
|  | CJJ81176_0233 | Hypothetical | 13.23 |
|  | CJJ81176_0241 | Hypothetical | 12.65 |
|  | CJJ81176_0084 | Hypothetical | 12.50 |
|  | CJJ81176_0234 | Hypothetical | 12.08 |
|  | CJJ81176_0594 | Hypothetical | 11.27 |
|  | CJJ81176_1062 | Hypothetical | 11.11 |
|  | CJJ81176_0951 | Hypothetical | 10.56 |
|  | CJJ81176_0368 | Hypothetical | 9.84 |
|  | CJJ81176_1324 | Hypothetical | 9.53 |
|  | CJJ81176_0231 | Hypothetical | 9.36 |
|  | CJJ81176_0440 | Hypothetical | 9.10 |
|  | CJJ81176_0950 | Hypothetical | 8.14 |
|  | CJJ81176_0948 | Hypothetical | 7.67 |
|  | CJJ81176_0438 | Hypothetical | 7.13 |
|  | CJJ81176_0752 | Hypothetical | 7.05 |
|  | CJJ81176_1457 | Hypothetical | 7.02 |
|  | CJJ81176_0065 | Hypothetical | 6.53 |
|  | CJJ81176_0751 | Hypothetical | 6.42 |
|  | CJJ81176_1458 | Hypothetical | 6.36 |
|  | CJJ81176_0847 | Hypothetical | 6.35 |
|  | CJJ81176_0242 | Hypothetical | 5.84 |
|  | CJJ81176_1617 | Hypothetical | 5.70 |
|  | CJJ81176_1257 | Hypothetical | 5.59 |
|  | CJJ81176_1658 | Hypothetical | 5.44 |
|  | CJJ81176_1540 | Hypothetical | 5.32 |
|  | CJJ81176_0740 | Hypothetical | 5.04 |
|  | CJJ81176_0593 | Hypothetical | 4.86 |
|  | CJJ81176_1059 | Hypothetical | 4.83 |
|  | CJJ81176_0045 | Hypothetical | 4.72 |
|  | CJJ81176_1493 | Hypothetical | 4.51 |
|  | CJJ81176_1443 | Hypothetical | 4.47 |
|  | CJJ81176_0596 | Hypothetical | 4.47 |
|  | CJJ81176_1355 | Hypothetical | 4.41 |
|  | CJJ81176_1022 | Hypothetical | 4.35 |
|  | CJJ81176_1572 | Hypothetical | 4.20 |
|  | CJJ81176_0947 | Hypothetical | 4.05 |
|  | CJJ81176_0078 | Hypothetical | 4.05 |
|  | CJJ81176_1358 | Hypothetical | 4.02 |
|  | CJJ81176_pVir0035 | Hypothetical | 26.36 |
|  | CJJ81176_pVir0044 | Hypothetical | 12.95 |
|  | CJJ81176_pVir0036 | Hypothetical | 9.82 |
|  | CJJ81176_pVir0046 | Hypothetical | 9.26 |
|  | CJJ81176_pVir0048 | Hypothetical | 5.88 |
|  | CJJ81176_pVir0037 | Hypothetical | 5.77 |
|  | CJJ81176_pVir0013 | Hypothetical | 5.06 |
|  | CJJ81176_pVir0024 | Hypothetical | 4.20 |
|  | CJJ81176_pVir0022 | Hypothetical | 4.18 |
|  | CJJ81176_pVir0014 | Hypothetical | 4.00 |

*p_adj_ < 0.05, a corrected p-value analogous to a false detection rate of < 5%.
