# Supplementary material for: The Complete Campylobacter jejuni Transcriptome during Colonization of a Natural Host Determined by RNAseq
Source: PLoS One. 2013 Aug 21;8(8):e73586. doi: 10.1371/journal.pone.0073586 (PMC3749233; doi:10.1371/journal.pone.0073586)
Supplement: Table S2 — Genes decreased in abundance in vivo compared to in vitro mid-log phase cultures. Listed are genes with decreased abundance during in vivo colonization compared to in vitro mid-exponential phase broth grown cultures, as determined by DESeq analysis (materials and methods). Only genes significantly differentially regulated (>4-fold difference in abundance, padj<0.05) are listed. padj<0.05, is a corrected p-value analogous to a false detection rate of < 5%. Genes are grouped by functional classification and by their C. jejuni 81-176 locus numbers and gene name or function. (DOCX) [file pone.0073586.s004.docx]

Table S2. Genes decreased in abundance *in vivo* compared to *in vitro* mid-log phase cultures.

| Function Classification | CJJ Locus Number | Gene Name / Function | Fold Change* |
| --- | --- | --- | --- |
| Biosynthetic Processes | CJJ81176_1590 | *hisF* | 5.13 |
|  | CJJ81176_1587 | *hisH* | 4.32 |
|  | CJJ81176_pTet0048 | *tetO* | 210.77 |
| Energy and Metabolism | CJJ81176_0625 | *fbaA* | 4.09 |
| Protease | CJJ81176_0384 | *ispA* | 4.28 |
| Signal Transduction | CJJ81176_1240 | Sensor histidine kinase | 4.16 |
| Other | CJJ81176_pTet0030 | *cpp33* | 387.10 |
|  | CJJ81176_0535 | Maf-like protein | 4.00 |
|  | CJJ81176_0315 | PEB3 | 19.16 |
| Hypothetical | CJJ81176_pVir0008 | Hypothetical | 6.93 |
|  | CJJ81176_pVir0009 | Hypothetical | 6.02 |
|  | CJJ81176_0204 | Hypothetical | 5.07 |
|  | CJJ81176_1657 | Hypothetical | 4.60 |

*p_adj_ < 0.05, a corrected p-value analogous to a false detection rate of < 5%.
