# Supplementary material for: The Complete Campylobacter jejuni Transcriptome during Colonization of a Natural Host Determined by RNAseq
Source: PLoS One. 2013 Aug 21;8(8):e73586. doi: 10.1371/journal.pone.0073586 (PMC3749233; doi:10.1371/journal.pone.0073586)
Supplement: Table S3 — Genes increased in abundance in vivo compared to in vitro stationary phase cultures. Listed are genes with increased abundance during in vivo colonization compared to in vitro stationary phase broth grown cultures, as determined by DESeq analysis (materials and methods). Only genes significantly differentially regulated (>4-fold difference in abundance, padj<0.05) are listed. padj<0.05, is a corrected p-value analogous to a false detection rate of < 5%. Genes are grouped by functional classification and by their C. jejuni 81-176 locus numbers and gene name or function. (DOCX) [file pone.0073586.s005.docx]

Table S3. Genes increased in abundance *in vivo* compared to *in vitro* stationary phase cultures.

| Function Classification | CJJ Locus Number | Gene Name / Function | Fold Change* |
| --- | --- | --- | --- |
| Biosynthetic Processes | CJJ81176_1113 | *cutE* | 4.59 |
|  | CJJ81176_1161 | CMP-Neu5Ac synthase | 5.05 |
|  | CJJ81176_1392 | *metC* | 8.12 |
| DNA Modification and Repair | CJJ81176_0229 | *rarA* | 5.12 |
|  | CJJ81176_1119 | *uvrD* | 4.52 |
|  | CJJ81176_1390 | Endoribonulcease | 8.03 |
| Energy and Metabolism | CJJ81176_0044 | *dsbB* | 8.04 |
|  | CJJ81176_0064 | Cytochrome c family | 20.99 |
|  | CJJ81176_0065 | Hypothetical *dsbB* Like | 31.66 |
|  | CJJ81176_0118 | *cioA* | 15.22 |
|  | CJJ81176_0119 | *cioB* | 17.95 |
|  | CJJ81176_0122 | *aspA* | 9.41 |
|  | CJJ81176_0194 | Cytochrome c family | 4.61 |
|  | CJJ81176_0382 | *cppA*-2 | 6.26 |
|  | CJJ81176_0403 | Sulphite oxidase | 14.00 |
|  | CJJ81176_0463 | *sdhA* | 19.08 |
|  | CJJ81176_0464 | *sdhB* | 22.27 |
|  | CJJ81176_0465 | *sdhC* | 11.45 |
|  | CJJ81176_0880 | *dsbA* | 5.10 |
|  | CJJ81176_0881 | *dsbB* | 4.10 |
|  | CJJ81176_1655 | Thioredoxin-like protein | 13.89 |
| Motility and Chemotaxis | CJJ81176_0273 | MCP protein | 5.94 |
| Nucleic Acid Biosynthesis | CJJ81176_0976 | *purH* | 5.75 |
|  | CJJ81176_1393 | *purB-2* | 7.95 |
| Ribosome and RNA Modification | CJJ81176_0003 | RNA pseudouridine synthase | 4.04 |
|  | CJJ81176_0004 | Metallo-beta-lactamase | 5.28 |
|  | CJJ81176_1269 | *pnp* | 4.43 |
|  | CJJ81176_1686 | *rplO* | 6.05 |
|  | CJJ81176_1687 | *rpsE* | 8.01 |
|  | CJJ81176_1688 | *rplR* | 6.65 |
|  | CJJ81176_1689 | *rplF* | 5.75 |
|  | CJJ81176_1690 | *rpsH* | 5.20 |
|  | CJJ81176_1691 | *rpsN* | 5.44 |
|  | CJJ81176_1692 | *rplE* | 7.45 |
|  | CJJ81176_1693 | *rplX* | 6.73 |
|  | CJJ81176_1694 | *rplN* | 7.05 |
|  | CJJ81176_1695 | *rpsQ* | 6.92 |
|  | CJJ81176_1696 | *rpmC* | 5.92 |
|  | CJJ81176_1697 | *rplP* | 7.14 |
|  | CJJ81176_1698 | *rpsC* | 6.28 |
|  | CJJ81176_1699 | *rplV* | 5.15 |
|  | CJJ81176_1700 | *rpsS* | 4.48 |
|  | CJJ81176_1701 | *rplB* | 4.06 |
| Stress Response | CJJ81176_1387 | *katA* | 46.11 |
|  | CJJ81176_1574 | *cgb* | 4.72 |
| Transport | CJJ81176_0123 | *dcuA* | 10.85 |
|  | CJJ81176_0211 | *cfbpA* | 24.63 |
|  | CJJ81176_0642 | *pstS* | 44.54 |
|  | CJJ81176_0643 | *pstC* | 31.31 |
|  | CJJ81176_0644 | *pstA* | 5.74 |
|  | CJJ81176_0750 | ABC-transporter periplasmic substrate-binding protein | 24.95 |
|  | CJJ81176_0753 | ABC transport permease | 11.85 |
|  | CJJ81176_0754 | ABC transport permease | 10.82 |
|  | CJJ81176_1351 | *ceuB* | 11.69 |
|  | CJJ81176_1352 | *ceuC* | 4.10 |
|  | CJJ81176_1391 | *dcuD* | 7.13 |
|  | CJJ81176_1569 | Peptide ABC transporter | 7.82 |
|  | CJJ81176_1601 | *chuA* | 10.54 |
|  | CJJ81176_1602 | *chuB* | 5.20 |
|  | CJJ81176_1603 | *chuC* | 6.79 |
|  | CJJ81176_1604 | *chuD* | 8.19 |
|  | CJJ81176_1619 | *exbB-2* | 43.35 |
|  | CJJ81176_1620 | *exbD* | 49.24 |
|  | CJJ81176_1621 | *tonB-2* | 39.69 |
|  | CJJ81176_1649 | FTR1 | 21.00 |
|  | CJJ81176_1650 | *p19* | 44.63 |
|  | CJJ81176_1652 | ABC transport permease | 24.75 |
|  | CJJ81176_1653 | ABC transport permease | 20.10 |
|  | CJJ81176_1654 | ABC transport ATP binding | 18.26 |
|  | CJJ81176_1685 | *secY* | 4.92 |
| Other | CJJ81176_0250 | Acetyltransferase | 6.35 |
|  | CJJ81176_0882 | Arylsulfate sulfotransferase | 5.59 |
|  | CJJ81176_1162 | Acetyltransferase | 4.24 |
|  | CJJ81176_1318 | HAD family phosphatase | 5.74 |
|  | CJJ81176_1388 | Ankryin repeat protein | 18.96 |
|  | CJJ81176_1394 | MmgE/PrpD family protein | 9.09 |
| Hypothetical | CJJ81176_0045 | Hypothetical | 15.76 |
|  | CJJ81176_0063 | Hypothetical | 20.74 |
|  | CJJ81176_0117 | Hypothetical | 12.34 |
|  | CJJ81176_0127 | Hypothetical | 5.83 |
|  | CJJ81176_0128 | Hypothetical | 5.23 |
|  | CJJ81176_0212 | hypothetical | 23.93 |
|  | CJJ81176_0266 | Hypothetical | 4.29 |
|  | CJJ81176_0402 | Hypothetical | 12.85 |
|  | CJJ81176_0522 | Hypothetical | 5.19 |
|  | CJJ81176_0586 | Hypothetical | 6.77 |
|  | CJJ81176_0751 | Hypothetical | 16.33 |
|  | CJJ81176_0752 | Hypothetical | 21.44 |
|  | CJJ81176_0954 | Hypothetical | 9.70 |
|  | CJJ81176_1062 | Hypothetical | 11.19 |
|  | CJJ81176_1087 | Hypothetical | 6.74 |
|  | CJJ81176_1118 | Hypothetical | 4.33 |
|  | CJJ81176_1386 | hypothetical | 30.08 |
|  | CJJ81176_1746 | Hypothetical | 7.06 |
|  | CJJ81176_pVir0006 | Hypothetical | 4.02 |

*p_adj_ < 0.05, a corrected p-value analogous to a false detection rate of < 5%.
