# Supplementary material for: The Complete Campylobacter jejuni Transcriptome during Colonization of a Natural Host Determined by RNAseq
Source: PLoS One. 2013 Aug 21;8(8):e73586. doi: 10.1371/journal.pone.0073586 (PMC3749233; doi:10.1371/journal.pone.0073586)
Supplement: Table S4 — Genes decreased in abundance in vivo compared to in vitro stationary phase cultures. Listed are genes with decreased abundance during in vivo colonization compared to in vitro stationary phase broth grown cultures, as determined by DESeq analysis (materials and methods). Only genes significantly differentially regulated (>4-fold difference in abundance, padj<0.05) are listed. padj<0.05, is a corrected p-value analogous to a false detection rate of < 5%. Genes are grouped by functional classification and by their C. jejuni 81-176 locus numbers and gene name or function. (DOCX) [file pone.0073586.s006.docx]

Table S4. Genes decreased in abundance *in vivo* compared to *in vitro* stationary phase cultures.

| Function Classification | CJJ Locus Number | Gene Name / Function | Fold Change* |
| --- | --- | --- | --- |
| Biosynthetic Processes | CJJ81176_1345 | *dxr* | 4.28 |
|  | CJJ81176_1495 | *putA* | 5.75 |
|  | CJJ81176_0600 | *ribB* | 4.69 |
|  | CJJ81176_0478 | *thiC* | 4.09 |
| DNA Modification and Repair | CJJ81176_0037 | *comEA* | 6.22 |
|  | CJJ81176_0816 | Mur ligase protein | 4.28 |
| Energy and Metabolism | CJJ81176_1214 | 2OG-Fe(II) oxygenase oxidoreductase | 4.00 |
|  | CJJ81176_1522 | *acs* | 12.15 |
|  | CJJ81176_0924 | *cstA* | 11.01 |
|  | CJJ81176_0075 | Cytochrome c family | 8.10 |
|  | CJJ81176_1384 | *fldA* | 4.92 |
|  | CJJ81176_1283 | *hydA* | 4.82 |
| Motility and Chemotaxis | CJJ81176_0572 | *flaG* | 4.71 |
|  | CJJ81176_0553 | *flgB* | 4.63 |
|  | CJJ81176_1204 | MCP | 5.05 |
| Ribosome and RNA Modification | CJJ81176_0189 | RNA methyltranscferase | 5.65 |
|  | CJJ81176_0129 | *rplU* | 5.44 |
|  | CJJ81176_0130 | *rpmA* | 6.22 |
|  | CJJ81176_0984 | *rpmH* | 5.46 |
|  | CJJ81176_0393 | *rpsU* | 33.96 |
| Stress | CJJ81176_0067 | Gamma-glutamyltransferase | 13.40 |
| Transport | CJJ81176_0912 | Amino acid carrier protein | 4.02 |
|  | CJJ81176_0235 | Citrate transporter | 9.12 |
|  | CJJ81176_0113 | *lctP* | 8.14 |
|  | CJJ81176_0929 | *pebC* | 5.18 |
|  | CJJ81176_1494 | *putP* | 5.52 |
|  | CJJ81176_0683 | Tripeptide transporter | 9.03 |
|  | CJJ81176_0682 | Tripeptide transporter | 8.99 |
|  | CJJ81176_0685 | Tripeptide transporter | 4.32 |
| Other | CJJ81176_0134 | Biotin protein ligase | 5.31 |
|  | CJJ81176_1348 | Fibronectin binding protein | 4.69 |
|  | CJJ81176_1192 | *gmk* | 4.42 |
|  | CJJ81176_0907 | HIT protein | 4.03 |
|  | CJJ81176_0094 | HmcD domain protein | 5.29 |
|  | CJJ81176_0091 | Lysine decarboxylase | 6.29 |
|  | CJJ81176_0996 | Putative periplasmic protein | 5.59 |
|  | CJJ81176_pTet0030 | *cpp33* | 15.28 |
|  | CJJ81176_pTet0048 | *tetO* | 203.66 |
| Hypothetical | CJJ81176_0923 | Hypothetical | 13.00 |
|  | CJJ81176_1363 | Hypothetical | 9.72 |
|  | CJJ81176_0765 | Hypothetical | 7.70 |
|  | CJJ81176_1457 | Hypothetical | 7.69 |
|  | CJJ81176_0231 | Hypothetical | 7.48 |
|  | CJJ81176_0593 | Hypothetical | 7.02 |
|  | CJJ81176_0024 | Hypothetical | 6.92 |
|  | CJJ81176_1257 | Hypothetical | 6.37 |
|  | CJJ81176_0207 | Hypothetical | 6.29 |
|  | CJJ81176_1358 | Hypothetical | 6.27 |
|  | CJJ81176_0414 | Hypothetical | 6.21 |
|  | CJJ81176_0945 | Hypothetical | 6.04 |
|  | CJJ81176_1347 | Hypothetical | 5.27 |
|  | CJJ81176_1617 | Hypothetical | 4.98 |
|  | CJJ81176_0484 | Hypothetical | 4.94 |
|  | CJJ81176_1683 | Hypothetical | 4.57 |
|  | CJJ81176_0577 | Hypothetical | 4.48 |
|  | CJJ81176_0367 | Hypothetical | 4.34 |
|  | CJJ81176_0448 | Hypothetical | 4.04 |

*p_adj_ < 0.05, a corrected p-value analogous to a false detection rate of < 5%.
