# Supplementary material for: The Complete Campylobacter jejuni Transcriptome during Colonization of a Natural Host Determined by RNAseq
Source: PLoS One. 2013 Aug 21;8(8):e73586. doi: 10.1371/journal.pone.0073586 (PMC3749233; doi:10.1371/journal.pone.0073586)
Supplement: Table S5 — Genes increased in abundance in vitro mid-log compared to in vitro stationary phase cultures. Listed are genes with increased abundance during in vitro mid-exponential phase broth grown cultures compared to in vitro stationary phase broth grown cultures, as determined by DESeq analysis (materials and methods). Only genes significantly differentially regulated (>4-fold difference in abundance, padj<0.05) are listed. padj<0.05, is a corrected p-value analogous to a false detection rate of < 5%. Genes are grouped by functional classification and by their C. jejuni 81-176 locus numbers and gene name or function. (DOCX) [file pone.0073586.s007.docx]

Table S5. Genes increased in abundance *in vitro* mid-log compared to *in vitro* stationary phase cultures.

| Function Classification | CJJ Locus Number | Gene Name / Function | Fold Change* |
| --- | --- | --- | --- |
| Acetyltransferase | CJJ81176_0250 | Acetyltransferase | 7.21 |
|  | CJJ81176_1162 | Acetyltransferase | 6.82 |
|  | CJJ81176_1323 | Acetyltransferase | 5.93 |
| Amino Acid Biosynthesis/Metabolism | CJJ81176_0251 | *argB* | 4.47 |
|  | CJJ81176_0252 | *argD* | 4.09 |
|  | CJJ81176_1625 | *aroC* | 5.48 |
|  | CJJ81176_0784 | *cysE* | 5.33 |
|  | CJJ81176_0871 | *folD* | 4.64 |
|  | CJJ81176_0613 | *folP* | 4.65 |
|  | CJJ81176_1588 | *hisA* | 11.22 |
|  | CJJ81176_1586 | *hisB* | 4.20 |
|  | CJJ81176_1587 | *hisH* | 8.21 |
|  | CJJ81176_1591 | *hisI* | 6.55 |
|  | CJJ81176_0185 | Homoserine dehydrogenase | 4.15 |
|  | CJJ81176_1392 | *metC* | 10.52 |
|  | CJJ81176_0877 | *pabA* | 4.55 |
| Biosynthetic Processes | CJJ81176_1319 | 3-oxoacyl synthase III | 8.06 |
|  | CJJ81176_0326 | *bioC* | 7.72 |
|  | CJJ81176_1161 | CMP-Neu5Ac synthetase | 5.13 |
|  | CJJ81176_0531 | *hemH* | 5.38 |
|  | CJJ81176_1152 | LOS biosynthesis glycosyltransferase | 8.01 |
|  | CJJ81176_0197 | *moaA* | 5.10 |
|  | CJJ81176_1350 | *pldA* | 4.21 |
|  | CJJ81176_0544 | Putative acyltransferase | 4.55 |
|  | CJJ81176_1436 | Putative glycosyl transferase | 4.12 |
|  | CJJ81176_1435 | Putative sugar transferase | 4.66 |
|  | CJJ81176_1151 | *waaM* | 4.66 |
| Capsule and Glycosylation | CJJ81176_1140 | *pglD* | 5.31 |
|  | CJJ81176_1146 | *pglH* | 5.51 |
|  | CJJ81176_1145 | *pglI* | 7.76 |
| Cell Wall and Cell Shape | CJJ81176_0303 | *mreB* | 5.58 |
|  | CJJ81176_0304 | *mreC* | 5.83 |
|  | CJJ81176_1672 | *murB* | 5.72 |
|  | CJJ81176_0536 | *pbpA* | 4.00 |
|  | CJJ81176_0859 | Putative transglycosylase | 5.67 |
| Chaperone | CJJ81176_0977 | DnaJ domain-containing protein | 7.52 |
| DNA Modification/Repair | CJJ81176_0622 | Non-specific endonuclease | 6.73 |
|  | CJJ81176_1454 | Putative DNA methyltransferase | 6.21 |
|  | CJJ81176_0777 | Type I restriction modification | 4.03 |
|  | CJJ81176_0780 | Type I site-specific deoxyribonuclease | 5.64 |
|  | CJJ81176_0068 | Type II restriction-modification | 8.70 |
|  | CJJ81176_1119 | *uvrD* | 11.84 |
| DNA Recombination/Replication | CJJ81176_1095 | *ctsT* | 5.05 |
|  | CJJ81176_pTet0045 | DNA topoisomerase III | 42.97 |
|  | CJJ81176_0662 | *dprA* | 4.93 |
|  | CJJ81176_0229 | *rarA* | 9.74 |
|  | CJJ81176_1474 | RecB-like protein | 6.70 |
|  | CJJ81176_0489 | *recG* | 7.67 |
|  | CJJ81176_pTet0031 | Single-stranded biding protein | 59.56 |
|  | CJJ81176_1240 | Sensor histidine kinase | 4.33 |
|  | CJJ81176_0391 | *tetR* | 4.30 |
| Energy and Metabolism | CJJ81176_0532 | Gfo/Idh/MocA family oxidoreductase | 10.02 |
|  | CJJ81176_0122 | *aspA* | 17.01 |
|  | CJJ81176_0118 | *cioA* | 4.96 |
|  | CJJ81176_0119 | *cioB* | 6.78 |
|  | CJJ81176_1032 | Cytochrome c biogenesis protein | 5.27 |
|  | CJJ81176_0194 | Cytochrome c family protein | 5.81 |
|  | CJJ81176_0625 | *fbaA* | 4.05 |
|  | CJJ81176_1503 | *fdhA* | 4.12 |
|  | CJJ81176_1502 | *fdhB* | 6.42 |
|  | CJJ81176_1501 | *fdhC* | 8.56 |
|  | CJJ81176_1427 | GDP-fucose synthetase | 6.59 |
|  | CJJ81176_1557 | *nuoH* | 4.58 |
|  | CJJ81176_1556 | *nuoI* | 4.08 |
|  | CJJ81176_1555 | *nuoJ* | 4.20 |
|  | CJJ81176_1553 | *nuoL* | 4.99 |
|  | CJJ81176_1552 | *nuoM* | 5.31 |
|  | CJJ81176_1551 | *nuoN* | 5.46 |
|  | CJJ81176_1200 | *petB* | 4.65 |
|  | CJJ81176_1199 | *petC* | 6.90 |
|  | CJJ81176_0571 | Putative carboxy-lyase, | 4.44 |
|  | CJJ81176_0463 | *sdhA* | 41.03 |
|  | CJJ81176_0464 | *sdhB* | 28.07 |
|  | CJJ81176_0465 | *sdhC* | 11.52 |
|  | CJJ81176_1655 | Thiredoxin,-like protein | 12.04 |
|  | CJJ81176_1636 | *tkt* | 6.03 |
|  | CJJ81176_1426 | *wcbK* | 5.81 |
| Motilty and Chemotaxis | CJJ81176_0342 | *fliH* | 4.06 |
|  | CJJ81176_0273 | MCP signal transduction protein | 10.73 |
|  | CJJ81176_1335 | Motility accessory factor | 7.35 |
| Nucleic Acid Biosynthesis | CJJ81176_0255 | Nicotinate phosphoribosyltransferase | 5.01 |
|  | CJJ81176_1393 | *purB-2* | 12.32 |
|  | CJJ81176_0976 | *purH* | 19.14 |
|  | CJJ81176_0978 | *purL* | 7.05 |
|  | CJJ81176_0218 | *purN* | 6.87 |
| Protease | CJJ81176_0842 | A24 family peptidase | 9.81 |
|  | CJJ81176_0872 | *lepB* | 6.73 |
|  | CJJ81176_0384 | *lspA* | 5.68 |
|  | CJJ81176_1117 | *pepF* | 5.24 |
|  | CJJ81176_0156 | Putative metalloprotease | 5.46 |
|  | CJJ81176_1086 | Putative zinc metalloprotease | 5.69 |
|  | CJJ81176_0106 | *sppA* | 9.30 |
| Ribosome and RNA Processing/Modification | CJJ81176_1269 | *pnp* | 4.28 |
|  | CJJ81176_0568 | *proS* | 4.11 |
|  | CJJ81176_1390 | Putative endoribonuclease | 7.61 |
|  | CJJ81176_0196 | Radical SAM domain-containing protein | 4.50 |
|  | CJJ81176_0007 | Ribosomal methyltransferase | 6.48 |
|  | CJJ81176_1627 | *rnh* | 6.48 |
|  | CJJ81176_1692 | *rplE* | 6.44 |
|  | CJJ81176_1689 | *rplF* | 5.97 |
|  | CJJ81176_1694 | *rplN* | 5.95 |
|  | CJJ81176_1686 | *rplO* | 7.94 |
|  | CJJ81176_1697 | *rplP* | 4.68 |
|  | CJJ81176_1688 | *rplR* | 7.79 |
|  | CJJ81176_1693 | *rplX* | 5.67 |
|  | CJJ81176_1696 | *rpmC* | 4.35 |
|  | CJJ81176_1698 | *rpsC* | 4.36 |
|  | CJJ81176_1687 | *rpsE* | 8.37 |
|  | CJJ81176_1690 | *rpsH* | 4.83 |
|  | CJJ81176_1691 | *rpsN* | 4.19 |
|  | CJJ81176_1695 | *rpsQ* | 5.33 |
|  | CJJ81176_0192 | *rsmE* | 8.94 |
|  | CJJ81176_0979 | *trmE* | 4.48 |
|  | CJJ81176_0844 | *truA* | 5.55 |
| Stress Response | CJJ81176_0382 | *cppA-2* | 6.92 |
| Transport | CJJ81176_0145 | Putative ExbD | 4.39 |
|  | CJJ81176_0701 | Putative potassium-transporting ATPase | 4.44 |
|  | CJJ81176_0214 | Putative transporter | 8.23 |
|  | CJJ81176_0750 | ABC transporter periplasmic | 4.86 |
|  | CJJ81176_1654 | ABC transporter, ATP-binding protein | 14.67 |
|  | CJJ81176_1652 | ABC transporter, permease protein | 22.38 |
|  | CJJ81176_1653 | ABC transporter, permease protein | 18.80 |
|  | CJJ81176_1052 | AcrB/AcrD/AcrF family protein | 6.41 |
|  | CJJ81176_0494 | Amino acid ABC transporter, ATP-binding protein | 5.18 |
|  | CJJ81176_0123 | *dcuA* | 15.49 |
|  | CJJ81176_1391 | *dcuD* | 10.90 |
|  | CJJ81176_1620 | *exbD* | 6.00 |
|  | CJJ81176_1649 | FTR1 family iron permease | 5.70 |
|  | CJJ81176_0209 | Iron ABC transporter, ATP binding subunit | 4.11 |
|  | CJJ81176_0211 | Iron ABC transporter, periplasmic iron-binding protein | 18.87 |
|  | CJJ81176_0210 | Iron ABC transporter, permease protein | 4.70 |
|  | CJJ81176_1033 | *livF* | 5.97 |
|  | CJJ81176_1034 | *livG* | 4.33 |
|  | CJJ81176_1036 | *livH* | 4.40 |
|  | CJJ81176_1035 | *livM* | 7.57 |
|  | CJJ81176_0322 | *modC* | 4.18 |
|  | CJJ81176_0637 | Outer membrane efflux protein | 5.69 |
|  | CJJ81176_0643 | *pstC* | 4.87 |
|  | CJJ81176_0642 | *pstS* | 5.83 |
|  | CJJ81176_1637 | Putative ABC transporter permease | 5.87 |
|  | CJJ81176_0965 | Putative permease | 4.47 |
|  | CJJ81176_1621 | Putative TonB-dependent receptor | 17.85 |
|  | CJJ81176_1685 | *secY* | 6.24 |
|  | CJJ81176_pVir0005 | *trbB* | 6.28 |
|  | CJJ81176_1147 | *wlaB* | 5.23 |
| Other | CJJ81176_0105 | Chlorohydrolase | 8.22 |
|  | CJJ81176_0154 | Putative pyrazinamidase/nicotinamidase | 4.59 |
|  | CJJ81176_0216 | PhnA domain-containing protein | 4.40 |
|  | CJJ81176_0315 | PEB3 | 64.25 |
|  | CJJ81176_0436 | GTP-binding protein | 5.03 |
|  | CJJ81176_0520 | Hit family protein | 4.25 |
|  | CJJ81176_0545 | *crcB* | 7.13 |
|  | CJJ81176_0841 | *uppS* | 7.31 |
|  | CJJ81176_0860 | YGGT family protein | 4.28 |
|  | CJJ81176_0919 | SCO1/SenC family protein | 4.64 |
|  | CJJ81176_1249 | M24/M37 family peptidase | 4.61 |
|  | CJJ81176_1318 | HAD family phosphatase | 12.57 |
|  | CJJ81176_1320 | Putative acyl carrier protein | 5.31 |
|  | CJJ81176_1383 | Putative lipoprotein | 7.89 |
|  | CJJ81176_1394 | MmgE/PrpD family protein | 17.65 |
|  | CJJ81176_1589 | HrgA protein | 13.57 |
|  | CJJ81176_pTet0016 | *cpp21* | 5.52 |
|  | CJJ81176_pTet0028 | *cmgB3/4* | 5.15 |
|  | CJJ81176_pTet0029 | *cpp32* | 14.48 |
|  | CJJ81176_pTet0030 | *cpp33* | 26.37 |
|  | CJJ81176_pTet0032 | *cpp35* | 114.69 |
|  | CJJ81176_pTet0033 | *cmgB5* | 19.85 |
|  | CJJ81176_pTet0034 | *cmgB6* | 6.34 |
|  | CJJ81176_pTet0036 | *cmgB8* | 178.44 |
|  | CJJ81176_pTet0037 | *cmgB9* | 105.51 |
|  | CJJ81176_pTet0038 | *cmgB10* | 81.76 |
|  | CJJ81176_pTet0039 | *cmgB11* | 5.25 |
|  | CJJ81176_pTet0040 | *cmgD4* | 79.43 |
|  | CJJ81176_pTet0041 | *cpp44* | 82.77 |
|  | CJJ81176_pTet0042 | *cpp45* | 85.63 |
|  | CJJ81176_pTet0043 | *cpp46* | 21.75 |
|  | CJJ81176_pTet0044 | *cpp47* | 213.61 |
|  | CJJ81176_pTet0046 | *cpp50* | 4.31 |
|  | CJJ81176_pVir0002 | VirB9 | 4.59 |
|  | CJJ81176_pVir0003 | VirB10 | 5.68 |
| Hypothetical | CJJ81176_0065 | Hypothetical | 5.06 |
|  | CJJ81176_0069 | Hypothetical | 4.85 |
|  | CJJ81176_0107 | Hypothetical | 4.80 |
|  | CJJ81176_0127 | Hypothetical | 6.48 |
|  | CJJ81176_0128 | Hypothetical | 7.92 |
|  | CJJ81176_0168 | Hypothetical | 4.41 |
|  | CJJ81176_0212 | Hypothetical | 10.34 |
|  | CJJ81176_0293 | Hypothetical | 4.12 |
|  | CJJ81176_0327 | Hypothetical | 5.30 |
|  | CJJ81176_0402 | Hypothetical | 6.72 |
|  | CJJ81176_0543 | Hypothetical | 6.35 |
|  | CJJ81176_0569 | Hypothetical | 4.04 |
|  | CJJ81176_0582 | Hypothetical | 5.38 |
|  | CJJ81176_0615 | Hypothetical | 8.12 |
|  | CJJ81176_0621 | Hypothetical | 7.10 |
|  | CJJ81176_0638 | Hypothetical | 4.49 |
|  | CJJ81176_0686 | Hypothetical | 4.98 |
|  | CJJ81176_0687 | Hypothetical | 6.72 |
|  | CJJ81176_0715 | Hypothetical | 4.15 |
|  | CJJ81176_0779 | Hypothetical | 5.51 |
|  | CJJ81176_0781 | Hypothetical | 4.55 |
|  | CJJ81176_0840 | Hypothetical | 4.83 |
|  | CJJ81176_0888 | Hypothetical | 4.72 |
|  | CJJ81176_1051 | Hypothetical | 4.12 |
|  | CJJ81176_1087 | Hypothetical | 11.89 |
|  | CJJ81176_1118 | Hypothetical | 7.75 |
|  | CJJ81176_1131 | Hypothetical | 5.39 |
|  | CJJ81176_1270 | Hypothetical | 4.04 |
|  | CJJ81176_1317 | Hypothetical | 7.17 |
|  | CJJ81176_1425 | Hypothetical | 4.14 |
|  | CJJ81176_1428 | Hypothetical | 4.93 |
|  | CJJ81176_1429 | Hypothetical | 8.07 |
|  | CJJ81176_1577 | Hypothetical | 4.57 |
|  | CJJ81176_1650 | Hypothetical | 36.42 |
|  | CJJ81176_1657 | Hypothetical | 7.32 |
|  | CJJ81176_pTet0035 | Hypothetical | 154.58 |
|  | CJJ81176_pVir0004 | Hypothetical | 5.94 |
|  | CJJ81176_pVir0006 | Hypothetical | 7.10 |
|  | CJJ81176_pVir0007 | Hypothetical | 4.34 |
|  | CJJ81176_pVir0008 | Hypothetical | 12.08 |
|  | CJJ81176_pVir0009 | Hypothetical | 9.26 |
|  | CJJ81176_pVir0028 | Hypothetical | 5.45 |
|  | CJJ81176_pVir0029 | Hypothetical | 6.37 |

*p_adj_ < 0.05, a corrected p-value analogous to a false detection rate of < 5%.
