# Supplementary material for: The Complete Campylobacter jejuni Transcriptome during Colonization of a Natural Host Determined by RNAseq
Source: PLoS One. 2013 Aug 21;8(8):e73586. doi: 10.1371/journal.pone.0073586 (PMC3749233; doi:10.1371/journal.pone.0073586)
Supplement: Table S6 — Genes decreased in abundance in vitro mid-log compared to in vitro stationary phase cultures. Listed are genes with decreased abundance during in vitro mid-exponential phase broth grown cultures compared to in vitro stationary phase broth grown cultures, as determined by DESeq analysis (materials and methods). Only genes significantly differentially regulated (>4-fold difference in abundance, padj<0.05) are listed. padj<0.05, is a corrected p-value analogous to a false detection rate of < 5%. Genes are grouped by functional classification and by their C. jejuni 81-176 locus numbers and gene name or function. (DOCX) [file pone.0073586.s008.docx]

Table S6. Genes decreased in abundance *in vitro* mid-log compared to *in vitro* stationary phase cultures.

| Function Classification | CJJ Locus Number | Gene Name / Function | Fold Change* |
| --- | --- | --- | --- |
| Amino acid biosynthesis | CJJ81176_0722 | *glnA* | 4.69 |
|  | CJJ81176_0602 | *ilvB* | 4.39 |
|  | CJJ81176_0017 | *leuA* | 9.08 |
|  | CJJ81176_0016 | *leuB* | 4.73 |
|  | CJJ81176_0014 | *leuD* | 4.07 |
|  | CJJ81176_1216 | *metE* | 18.48 |
|  | CJJ81176_1217 | *metF* | 10.26 |
|  | CJJ81176_0023 | *metX* | 5.08 |
|  | CJJ81176_1495 | *putA* | 7.97 |
|  | CJJ81176_1494 | *putP* | 5.63 |
| ATP Synthesis | CJJ81176_1219 | *atpB* | 4.40 |
|  | CJJ81176_0943 | *atpE* | 5.44 |
| Biosynthetic Processes | CJJ81176_0468 | *acpP* | 4.23 |
|  | CJJ81176_1346 | *cdsA* | 4.85 |
|  | CJJ81176_1345 | *dxr* | 5.42 |
|  | CJJ81176_1310 | *flmA* | 6.64 |
|  | CJJ81176_0010 | Putative LOS core biosynthesis protien | 5.31 |
|  | CJJ81176_0600 | *ribB* | 9.68 |
|  | CJJ81176_0478 | *thiC* | 10.46 |
| Capsule and Glycosylation | CJJ81176_1412 | *kpsS* | 5.58 |
| Cell Division | CJJ81176_0816 | Mur ligase family protein | 13.20 |
| Chaperone | CJJ81176_0537 | *clpB* | 14.55 |
|  | CJJ81176_1243 | *dnaJ1* | 9.60 |
|  | CJJ81176_0775 | *dnaK* | 4.48 |
|  | CJJ81176_1234 | *groEL* | 13.83 |
|  | CJJ81176_1233 | *groES* | 20.43 |
| Energy and Metabolism | CJJ81176_1214 | 2-OGFe(II) oxygenase family oxidoreductase | 75.05 |
|  | CJJ81176_0852 | *acnB* | 6.89 |
|  | CJJ81176_1522 | *acs* | 31.89 |
|  | CJJ81176_0924 | *cstA* | 22.72 |
|  | CJJ81176_0885 | Cytochrome C | 6.79 |
|  | CJJ81176_0075 | Cytochrome c family protein | 13.63 |
|  | CJJ81176_0884 | Cytochrome c family protein | 6.78 |
|  | CJJ81176_0067 | *ggt* | 46.60 |
|  | CJJ81176_0439 | Putative oxidoreductase | 4.11 |
|  | CJJ81176_0476 | *rpe* | 4.03 |
|  | CJJ81176_0932 | *rpiB* | 5.23 |
|  | CJJ81176_0183 | *trx* | 7.03 |
| Motility and Chemotaxis | CJJ81176_1204 | *cetB* | 4.05 |
|  | CJJ81176_0931 | *cheB* | 4.00 |
|  | CJJ81176_1136 | *cheY* | 4.32 |
|  | CJJ81176_0743 | *flaC* | 6.36 |
|  | CJJ81176_0572 | *flaG* | 18.85 |
|  | CJJ81176_0720 | Flagellar basalbody rod protein | 8.75 |
|  | CJJ81176_1339 | Flagellin | 4.71 |
|  | CJJ81176_0553 | *flgB* | 11.63 |
|  | CJJ81176_0552 | *flgC* | 6.04 |
|  | CJJ81176_0080 | *flgD* | 8.43 |
|  | CJJ81176_0710 | *flgH* | 8.60 |
|  | CJJ81176_1459 | *flgK* | 5.09 |
|  | CJJ81176_1455 | *flgL* | 11.75 |
|  | CJJ81176_0894 | *flgL* | 6.79 |
|  | CJJ81176_0573 | *fliD* | 4.88 |
|  | CJJ81176_0551 | *fliE* | 9.83 |
|  | CJJ81176_1205 | MCP | 5.21 |
|  | CJJ81176_0271 | MCP signal transduction protein | 16.46 |
| Nucleic Acid Biosynthesis/Metabolism | CJJ81176_1266 | *purD* | 7.34 |
|  | CJJ81176_0738 | Transthyretinlike protein | 4.93 |
| Protein Biosynthesis/Modification | CJJ81176_0134 | Biotinprotein ligase | 4.34 |
|  | CJJ81176_1212 | *gatB* | 4.01 |
|  | CJJ81176_0601 | GatB/Yqey family protein | 6.33 |
| Ribosome and RNA Processing | CJJ81176_0020 | 7-cyano-7-deazaguanine reductase | 4.64 |
|  | CJJ81176_0129 | *rplU* | 8.56 |
|  | CJJ81176_0130 | *rpmA* | 8.93 |
|  | CJJ81176_0475 | *rpmB* | 4.17 |
|  | CJJ81176_0984 | *rpmH* | 4.02 |
|  | CJJ81176_0892 | *rpsO* | 6.13 |
|  | CJJ81176_1598 | *rpsT* | 4.85 |
|  | CJJ81176_0393 | *rpsU* | 29.37 |
| Stress Response | CJJ81176_0356 | *ahpC* | 4.79 |
|  | CJJ81176_0774 | *grpE* | 8.35 |
|  | CJJ81176_1387 | *katA* | 6.66 |
|  | CJJ81176_0205 | *sodB* | 4.50 |
|  | CJJ81176_0800 | *tpx* | 4.10 |
| Transcription | CJJ81176_0773 | *hrcA* | 7.44 |
|  | CJJ81176_1244 | *merR* | 7.12 |
|  | CJJ81176_0485 | *nusA* | 4.35 |
|  | CJJ81176_0671 | Response regulator/GGDEF domaincontaining protein | 4.19 |
|  | CJJ81176_1542 | Transcriptional regulator | 10.13 |
| Transport | CJJ81176_0087 | 2-oxoglutarate/malate translocator | 14.37 |
|  | CJJ81176_0089 | 2-oxoglutarate/malate translocator | 6.73 |
|  | CJJ81176_1638 | ABC transporter, ATPbinding protein | 5.01 |
|  | CJJ81176_0912 | Amino acid carrier protein | 4.44 |
|  | CJJ81176_0836 | Amino acidbinding protein | 7.44 |
|  | CJJ81176_0086 | Anion transporter | 16.84 |
|  | CJJ81176_0085 | Anion transporter | 15.79 |
|  | CJJ81176_0088 | Anion transporter | 12.34 |
|  | CJJ81176_1353 | *ceuD* | 4.38 |
|  | CJJ81176_0235 | Citrate transporter | 7.69 |
|  | CJJ81176_0683 | Di/tripeptide transporter | 40.08 |
|  | CJJ81176_0682 | Di/tripeptide transporter | 23.52 |
|  | CJJ81176_1354 | Enterochelin ABC transporter, | 4.08 |
|  | CJJ81176_0113 | *lctP* | 8.20 |
|  | CJJ81176_0866 | Major facilitator family protein | 4.53 |
|  | CJJ81176_1646 | *nhaA* | 5.68 |
|  | CJJ81176_0926 | PEB1 | 4.14 |
|  | CJJ81176_0928 | *pebA* | 4.42 |
|  | CJJ81176_0929 | *pebC* | 8.19 |
|  | CJJ81176_1060 | Putative transmembrane transport protein | 4.48 |
|  | CJJ81176_1191 | TAT protein translocase | 4.39 |
| Other | CJJ81176_0013 | Acetyltransferase | 4.48 |
|  | CJJ81176_0091 | Lysine decarboxylase protein | 4.67 |
|  | CJJ81176_0108 | Hemerythrin nonheme iron protein | 9.77 |
|  | CJJ81176_0394 | Colicin E1 immunity protein | 26.71 |
|  | CJJ81176_0524 | Putative periplasmic protein | 34.41 |
|  | CJJ81176_0525 | Putative periplasmic protein | 14.20 |
|  | CJJ81176_0526 | Putative periplasmic protein | 7.37 |
|  | CJJ81176_0527 | Putative periplasmic protein | 18.45 |
|  | CJJ81176_0530 | Putative outermembrane protein | 5.13 |
|  | CJJ81176_0597 | Putative transcriptional regulator | 16.58 |
|  | CJJ81176_0609 | *nidH* | 4.79 |
|  | CJJ81176_0760 | Hemagglutination domaincontaining protein | 5.69 |
|  | CJJ81176_0793 | NLPA family lipoprotein | 5.67 |
|  | CJJ81176_0818 | Preventhostdeath family protein | 7.60 |
|  | CJJ81176_0922 | Thioesterase family protein | 4.39 |
|  | CJJ81176_0988 | Thioesterase family protein | 4.29 |
|  | CJJ81176_0990 | Putative periplasmic protein | 17.85 |
|  | CJJ81176_0991 | Putative periplasmic protein | 33.35 |
|  | CJJ81176_0992 | Putative periplasmic protein | 11.56 |
|  | CJJ81176_0993 | Putative periplasmic protein | 7.87 |
|  | CJJ81176_1045 | Putative lipoprotein | 8.70 |
|  | CJJ81176_1053 | Adenylosuccinate lyase | 6.26 |
|  | CJJ81176_1198 | Putative phospholipid synthase | 7.23 |
|  | CJJ81176_1215 | NLPA family lipoprotein | 37.89 |
|  | CJJ81176_1222 | Putative lipoprotein | 4.24 |
|  | CJJ81176_1348 | Putative fibronectin binding protein | 6.54 |
|  | CJJ81176_1388 | Ankyrin repeatcontaining protein | 4.44 |
|  | CJJ81176_1623 | Putative periplasmic protein | 16.65 |
|  | CJJ81176_1741 | Putative periplasmic protein | 15.11 |
|  | CJJ81176_pTet0004 | *cpp8* | 8.47 |
|  | CJJ81176_pTet0008 | *cpp12* | 14.86 |
|  | CJJ81176_pTet0009 | *cpp13* | 5.13 |
|  | CJJ81176_pTet0018 | *cpp23* | 8.71 |
|  | CJJ81176_pVir0025 | Para protein | 12.13 |
| Hypothetical | CJJ81176_0024 | Hypothetical | 13.98 |
|  | CJJ81176_0078 | Hypothetical | 11.67 |
|  | CJJ81176_0083 | Hypothetical | 6.05 |
|  | CJJ81176_0084 | Hypothetical | 8.53 |
|  | CJJ81176_0093 | Hypothetical | 5.29 |
|  | CJJ81176_0100 | Hypothetical | 8.58 |
|  | CJJ81176_0109 | Hypothetical | 6.32 |
|  | CJJ81176_0231 | Hypothetical | 66.98 |
|  | CJJ81176_0232 | Hypothetical | 7.42 |
|  | CJJ81176_0233 | Hypothetical | 12.20 |
|  | CJJ81176_0234 | Hypothetical | 11.15 |
|  | CJJ81176_0241 | Hypothetical | 10.01 |
|  | CJJ81176_0287 | Hypothetical | 8.53 |
|  | CJJ81176_0367 | Hypothetical | 4.95 |
|  | CJJ81176_0368 | Hypothetical | 5.35 |
|  | CJJ81176_0414 | Hypothetical | 16.64 |
|  | CJJ81176_0438 | Hypothetical | 8.25 |
|  | CJJ81176_0440 | Hypothetical | 8.19 |
|  | CJJ81176_0445 | Hypothetical | 9.84 |
|  | CJJ81176_0448 | Hypothetical | 10.04 |
|  | CJJ81176_0467 | Hypothetical | 7.84 |
|  | CJJ81176_0482 | Hypothetical | 6.25 |
|  | CJJ81176_0484 | Hypothetical | 8.48 |
|  | CJJ81176_0523 | Hypothetical | 22.30 |
|  | CJJ81176_0564 | Hypothetical | 5.60 |
|  | CJJ81176_0577 | Hypothetical | 4.24 |
|  | CJJ81176_0593 | Hypothetical | 32.48 |
|  | CJJ81176_0594 | Hypothetical | 35.36 |
|  | CJJ81176_0596 | Hypothetical | 5.77 |
|  | CJJ81176_0747 | Hypothetical | 9.21 |
|  | CJJ81176_0758 | Hypothetical | 37.89 |
|  | CJJ81176_0759 | Hypothetical | 11.35 |
|  | CJJ81176_0761 | Hypothetical | 6.90 |
|  | CJJ81176_0765 | Hypothetical | 26.35 |
|  | CJJ81176_0847 | Hypothetical | 12.98 |
|  | CJJ81176_0867 | Hypothetical | 8.39 |
|  | CJJ81176_0886 | Hypothetical | 4.68 |
|  | CJJ81176_0923 | Hypothetical | 20.36 |
|  | CJJ81176_0945 | Hypothetical | 16.48 |
|  | CJJ81176_0946 | Hypothetical | 10.53 |
|  | CJJ81176_0947 | Hypothetical | 6.79 |
|  | CJJ81176_0948 | Hypothetical | 17.35 |
|  | CJJ81176_0949 | Hypothetical | 50.42 |
|  | CJJ81176_0951 | Hypothetical | 5.94 |
|  | CJJ81176_0952 | Hypothetical | 14.43 |
|  | CJJ81176_0953 | Hypothetical | 5.28 |
|  | CJJ81176_0996 | Hypothetical | 15.27 |
|  | CJJ81176_1006 | Hypothetical | 6.35 |
|  | CJJ81176_1007 | Hypothetical | 7.24 |
|  | CJJ81176_1022 | Hypothetical | 14.10 |
|  | CJJ81176_1044 | Hypothetical | 4.14 |
|  | CJJ81176_1059 | Hypothetical | 4.23 |
|  | CJJ81176_1257 | Hypothetical | 33.95 |
|  | CJJ81176_1324 | Hypothetical | 4.76 |
|  | CJJ81176_1344 | Hypothetical | 4.28 |
|  | CJJ81176_1347 | Hypothetical | 18.99 |
|  | CJJ81176_1355 | Hypothetical | 4.44 |
|  | CJJ81176_1358 | Hypothetical | 24.07 |
|  | CJJ81176_1363 | Hypothetical | 10.95 |
|  | CJJ81176_1443 | Hypothetical | 9.19 |
|  | CJJ81176_1457 | Hypothetical | 51.49 |
|  | CJJ81176_1458 | Hypothetical | 23.62 |
|  | CJJ81176_1461 | Hypothetical | 5.44 |
|  | CJJ81176_1462 | Hypothetical | 4.09 |
|  | CJJ81176_1487 | Hypothetical | 4.06 |
|  | CJJ81176_1540 | Hypothetical | 15.51 |
|  | CJJ81176_1573 | Hypothetical | 8.76 |
|  | CJJ81176_1617 | Hypothetical | 27.12 |
|  | CJJ81176_1622 | Hypothetical | 5.50 |
|  | CJJ81176_1647 | Hypothetical | 4.94 |
|  | CJJ81176_1648 | Hypothetical | 5.50 |
|  | CJJ81176_1742 | Hypothetical | 67.66 |
|  | CJJ81176_1744 | Hypothetical | 5.16 |
|  | CJJ81176_1747 | Hypothetical | 4.06 |
|  | CJJ81176_pTet0019 | Hypothetical | 4.90 |
|  | CJJ81176_pTet0021 | Hypothetical | 19.03 |
|  | CJJ81176_pTet0022 | Hypothetical | 7.73 |
|  | CJJ81176_pVir0013 | Hypothetical | 11.11 |
|  | CJJ81176_pVir0014 | Hypothetical | 6.91 |
|  | CJJ81176_pVir0022 | Hypothetical | 4.15 |
|  | CJJ81176_pVir0026 | Hypothetical | 5.99 |
|  | CJJ81176_pVir0035 | Hypothetical | 19.04 |
|  | CJJ81176_pVir0036 | Hypothetical | 11.08 |
|  | CJJ81176_pVir0037 | Hypothetical | 7.89 |
|  | CJJ81176_pVir0044 | Hypothetical | 8.21 |
|  | CJJ81176_pVir0046 | Hypothetical | 7.07 |
|  | CJJ81176_pVir0047 | Hypothetical | 4.16 |
|  | CJJ81176_pVir0048 | Hypothetical | 8.11 |
|  | CJJ81176_pVir0050 | Hypothetical | 6.78 |

*p_adj_ < 0.05, a corrected p-value analogous to a false detection rate of < 5%.
