# Supplementary material for: The Complete Campylobacter jejuni Transcriptome during Colonization of a Natural Host Determined by RNAseq
Source: PLoS One. 2013 Aug 21;8(8):e73586. doi: 10.1371/journal.pone.0073586 (PMC3749233; doi:10.1371/journal.pone.0073586)
Supplement: Table S7 — Non-coding RNAs that align to pTet. Location of non-coding RNA species identified by RNAseq that map to the pTET plasmid. (DOCX) [file pone.0073586.s009.docx]

Table S7. Non-coding RNAs that align to pTet.

| ncRNA ID | Location on pTET |
| --- | --- |
| pnc 1 | 25973..26068 |
| pnc 2 | 3867..4028 |
| pnc 3 | 4236..4364 |
| pnc 4 | 26466..26591 |
| pnc 5 | 28323..28891 |
| pnc 6 | 31984..32443 |
| pnc 7 | 35822..36033 |
| pnc 8 | 36437..36622 |
| pnc 9 | 39476..40841 |
| pnc 10 | 40842..42024 |
| pnc 11 | 42060..42438 |
| pnc 12 | 5150..6110 |
| pnc 13 | 14408..15288 |
| pnc 14 | 16359..16557 |
| pnc 15 | 17508..18399 |
| pnc 16 | 21575..22878 |
| pnc 17 | 24727..24829 |
| pnc 18 | 25268..25894 |
