# Supplementary material for: The Complete Campylobacter jejuni Transcriptome during Colonization of a Natural Host Determined by RNAseq
Source: PLoS One. 2013 Aug 21;8(8):e73586. doi: 10.1371/journal.pone.0073586 (PMC3749233; doi:10.1371/journal.pone.0073586)
Supplement: Table S8 — Primers used in qRT-PCR experiments. Listed are the primer sets, along with their sequence, used in qRT-PCR experiments. Each primer set is annotated by which gene it will amplify. (DOCX) [file pone.0073586.s010.docx]

Table S8. Primers used in qRT-PCR experiments.

| Primer | Sequence |
| --- | --- |
| *gyrA* RT F | GCTGTAGGTATGGCGACAAAC |
| *gyrA* RT R | CCTGTTGGAAAATCTGGACC |
| *katA* RT-F | GCGGATGAAGAATGTCGGAGTG |
| *katA* RT-R | GCGGATGAAGAATGTCGGAGTG |
| *cjaC* RT-F | GGGTTGAAACCAATTTCGATGG |
| *cjaC* RT-R | CTTTGTCTTCTTTCGTCAGTTGCG |
| *Cjj0315* RT-F | AATCTTTACGGACCAGGTGG |
| *Cjj0315* RT-R | GCCTTTTCAAACCAAGTCGC |
| *ccoN* RT-F | TGTTTGGCATTGTTGGTATGG |
| *ccoN* RT-R | TCACACCTGAAGTATGAAGTGGTC |
| *Cjj0067* RT-F | CAATCACGCTAAGTTTTGGTG |
| *Cjj0067* RT-R | GCATCTATGGCATTTCCGC |
